# Genetic variation in Tertiary relics: The case of eastern‐Mediterranean *Abies* (Pinaceae)

**DOI:** 10.1002/ece3.3519

**Published:** 2017-10-22

**Authors:** Matúš Hrivnák, Ladislav Paule, Diana Krajmerová, Şemsettin Kulaç, Hakan Şevik, İbrahim Turna, Irina Tvauri, Dušan Gömöry

**Affiliations:** ^1^ Technical University in Zvolen Zvolen Slovakia; ^2^ Faculty of Forestry Düzce University Düzce Turkey; ^3^ Faculty of Engineering and Architecture Kastamonu University Kastamonu Turkey; ^4^ Faculty of Forestry Karadeniz Technical University Trabzon Turkey; ^5^ Scientific‐Research Center of Agriculture Tbilisi Georgia; ^6^ Vasil Gulisashvili Forest Institute Agricultural University of Georgia Tbilisi Georgia

**Keywords:** *Abies bornmuelleriana*, *Abies cilicica*, *Abies equi‐trojani*, *Abies nordmanniana*, Approximate Bayesian Computation, diversity, phylogeny

## Abstract

The eastern‐Mediterranean *Abies* taxa, which include both widely distributed species and taxa with minuscule ranges, represent a good model to study the impacts of range size and fragmentation on the levels of genetic diversity and differentiation. To assess the patterns of genetic diversity and phylogenetic relationships among eastern‐Mediterranean *Abies* taxa, genetic variation was assessed by eight nuclear microsatellite loci in 52 populations of *Abies* taxa with a focus on those distributed in Turkey and the Caucasus. Both at the population and the taxon level, the subspecies or regional populations of *Abies nordmanniana* s.l. exhibited generally higher allelic richness, private allelic richness, and expected heterozygosity compared with *Abies cilicica* s.l. Results of both the structure analysis and distance‐based approaches showed a strong differentiation of the two *A. cilicica* subspecies from the rest as well as from each other, whereas the subspecies of *A. nordmanniana* were distinct but less differentiated. ABC simulations were run for a set of scenarios of phylogeny and past demographic changes. For *A*. ×*olcayana,* the simulation gave a poor support for the hypothesis of being a taxon resulting from a past hybridization, the same is true for *Abies equi‐trojani*: both they represent evolutionary branches of *Abies bornmuelleriana*.

## 1 INTRODUCTION

Even though theoretical population‐genetic models predict that range fragmentation and small population size generally result in low intrapopulation diversity and high differentiation (Kimura & Crow, 1964), empirical data show that the extent of the erosion of gene diversity and the levels of genetic divergence depends from the degree of fragmentation related to gene flow rate, dispersal mechanisms, demographic structure, and other factors (Bialozyt, Ziegenhagen, & Petit, 2006; Ellstrand & Elam, 1993; Young, Boyle, & Brown, 1996). In the case of sympatric or parapatric species complexes, the situation may further be complicated by hybridization and introgression among interfertile taxa (Bouillé, Senneville, & Bousquet, 2011). Interspecific gene flow is relevant not only for genetic variation levels but also for maintaining species integrity (Petit & Excoffier, 2009).

Fir (*Abies* Mill.) taxa in the Mediterranean basin represent a good model to study the effects of range fragmentation and evolutionary reticulation. The genus itself is the second‐largest in the family Pinaceae with the number of species varying between 48 and 59 depending on particular taxonomic revision (e.g., Farjon & Rushforth, 1989; Liu, 1971). The Mediterranean firs are classified into sections *Abies* and *Piceaster*. Recent molecular evidence suggests that the genus is monophyletic (Xiang, Xiang, Guo, & Zhang, 2009). The genus itself is supposed to have originated in northern Eurasia in the late Cretaceous (Xiang, Cao, & Zhou, 2007), but the divergence of the extant taxa occurred much later: the sections of the Mediterranean firs diverged in the Miocene (Aguirre‐Planter et al., 2012), whereas diversification of species within the section *Abies* occurred in the late Pliocene and early Pleistocene (Liu, 1971).

Four commonly recognized *Abies* species occur in eastern‐Mediterranean and Ponthic area (Farjon & Rushforth, 1989; Liu, 1971). *A. alba* Mill., *A. cephalonica* Loud., and *A. nordmanniana* (Steven) Spach are unanimously classified into the section *Abies*. *A. cilicica* (Antoine & Kotschy) Carr. with two subspecies (subsp. *cilicica* and subsp. *isaurica*) was assigned by Liu (1971) to section *Piceaster*, but Farjon and Rushforth (1989) placed it to section *Abies*. Additionally, there are *A. equi‐trojani* Asch. et Sint. ex Boiss. and *A. bornmuelleriana* Mattf., whose taxonomical status is controversial. They were described by Mattfeld, Bornmüller, and von Handel‐Mazzetti (1925) as separate species but later revised as subspecies of *A. nordmanniana* (Coode & Cullen, 1965), which is currently the most‐accepted view. Euro+Med PlantBase (Euro+Med, 2006) and IUCN (Knees & Gardner, 2011) treat *A. bornmuelleriana* as a synonym to *A. nordmanniana* subsp. *equi‐trojani*. In local studies, they still continue to be commonly referred to as separate species (e.g., Bergmann, Hosius, & Leinemann, 2013; Liepelt, Mayland‐Quellhorst, Lahme, & Ziegenhagen, 2010; Linares, 2011; Scaltsoyiannes, Tsaktsira, & Drouzas, 1999). In older literature, both species were suspected to be of hybrid origin (Klaehn & Winieski, 1962). Another hybrid taxon is *A. borisii‐regis* Mattf., located in Greece between the ranges of *A. alba* and *A. cephalonica* (Bella, Liepelt, Parducci, & Drouzas, 2015; Krajmerová et al., 2016), which is recognized by Euro+Med PlantBase as a separate species (Euro+Med, 2006). Finally, Ata and Merev (1987) described *A*. ×*olcayana* as a natural hybrid between *A. equi‐trojani* and *A. bornmuelleriana*. For brevity, we refer to subspecies of *A. nordmanniana* and *A. cilicica* as separate species in further text.

*Abies equi‐trojani*, the westernmost subspecies of *A. nordmanniana*, has a very small distribution range. According to Coode and Cullen (1965), it grows only on the Kazdağı Mountain (Mount Ida), a massif with area of approximately 700 km^2^ located in northwestern Turkey, close to ruins of Troy (hence the name of the subspecies). Distribution area of *A. bornmuelleriana* is located south from the Black Sea, mainly in the Köroğlu, Ilgaz, and Küre Mountains. Finally, the nominate subspecies *A. nordmanniana* subsp. *nordmanniana* (further *A. nordmanniana s.s*.) occurs near the southeastern and eastern coast of the Black Sea, from the Pontic Mountains to the western part of the Greater Caucasus. Far south from the *A. nordmanniana* populations, *A. cilicica* subsp. *cilicica* (further *A. cilicica s.s*.) grows in Central Taurus, Anti‐Taurus (Aladağlar), and the Amanos Mountains in Turkey, the Alawi Mountains in Syria and the Northern Mount Lebanon Range in Lebanon. Area of *A. cilicica* subsp. *isaurica* is located in the Western Taurus in Turkey.

**Figure 1:**
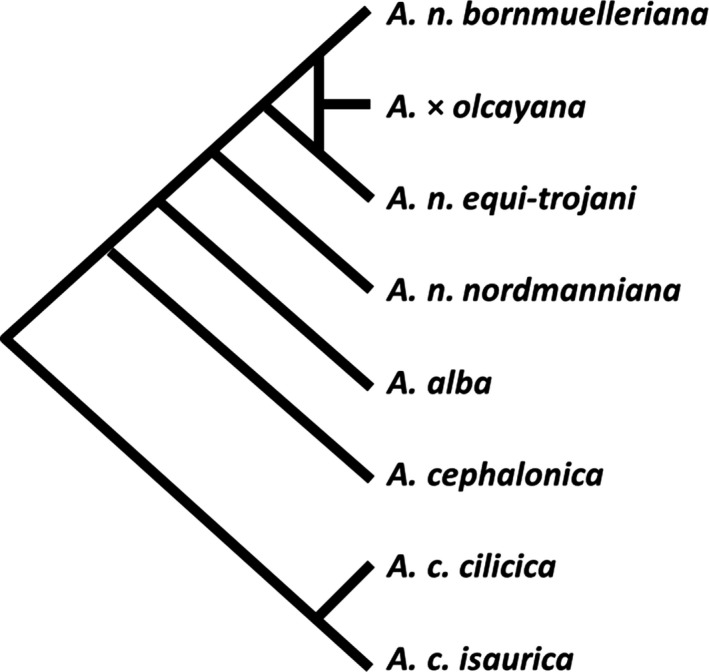
Scheme of speciation sequence in eastern‐Mediterranean *Abies* based on Linares (2011) with modification

Speciation sequence of fir species in the eastern‐Mediterranean region is not clearly resolved. Phylogenetic studies of the genus *Abies* that included *A. nordmanniana* and *A. cilicica* (Semerikova & Semerikov, 2014; Xiang et al., 2009, 2015) were typically restricted to the species level and did not distinguish among subspecies. In regional studies of genetic variation among Mediterranean firs, both those using traditional tools such as morphometry, allozymes, and terpenes (Bagci & Babaç, 2003; Fady, Arbez, & Marpeau, 1992; Fady & Conkle, 1993; Scaltsoyiannes et al., 1999) and the more recent ones employing molecular markers (Bergmann et al., 2013; Liepelt et al., 2010; Parducci & Szmidt, 1999), each taxon was mostly represented by one or two populations only, without distinguishing between the subspecies of *A. cilicica*, or studies focused on one species only or covered just a part of distribution ranges (Awad, Fady, Khater, Roig, & Cheddadi, 2014; Hansen, Kjær, & Vendramin, 2005; Sękiewicz et al., 2015). Based on fossil evidence, biogeography and marker studies, Linares (2011) suggested a picture of speciation and migration, which is schematically displayed in Figure 1. Several details of this scheme show discrepancies with fossil evidence as interpreted by Palamarev (1989) and organellar‐DNA variation patterns (e.g., Jaramillo‐Correa, Aguirre‐Planter, Eguiarte, Khasa, & Bousquet, 2013; Semerikova & Semerikov, 2014; Ziegenhagen, Fady, Kuhlenkamp, & Liepelt, 2005).

The main aim of this work was assessing the patterns of genetic diversity and phylogenetic relationships among eastern‐Mediterranean *Abies* taxa with a focus on *A. nordmanniana s.l*. and *A. cilicica s.l*. with a dense coverage that would allow better delineation of distribution ranges of each taxon, as they are disputed in some cases. In order to achieve this, populations covering major parts of the distribution ranges of *A. nordmanniana* and *A. cilicica* in Asia Minor and eastern Caucasus were sampled. Populations of the closely related European species *A. alba* and *A. cephalonica* at the eastern limit of their distribution were also included in order to set the results into larger geographical context of the eastern‐Mediterranean area. Some taxa that we studied are not recognized by the state‐of‐the‐art checklists and there is none or few information on their genetic structure, but they can be clearly geographically delineated and their names are widely used by local botanists; this is why they were of interest. The divergence of genetic lineages leading to recent *A. cilicica*,* A. cephalonica,* and *A. alba* occurred in the Miocene or even earlier (Linares, 2011; Palamarev, 1989). Therefore, we considered the reconstruction of the complete demographic history of the studied species complex beyond the reach of our possibilities with the type of data and the number of loci available. Instead, we focused on taxa within *A. nordmanniana* s.l. and *A. cilicica* s.l. In particular, we addressed the following questions:

- Is *A*. × *olcayana* a hybrid between *A. equi‐trojani* and *A. bornmuelleriana* (as suggested by Ata & Merev, 1987)?
- Is *A. equi‐trojani* a hybrid between *A. alba* and *A. bornmuelleriana* (as suggested by Klaehn & Winieski, 1962)?
- What is the realistic phylogenetic scenario for taxa or regional populations within *A. nordmanniana s.l*. and *A. cilicica s.l*.?

## 2 MATERIAL AND METHODS

### 2.1 Plant material

**Figure 2:**
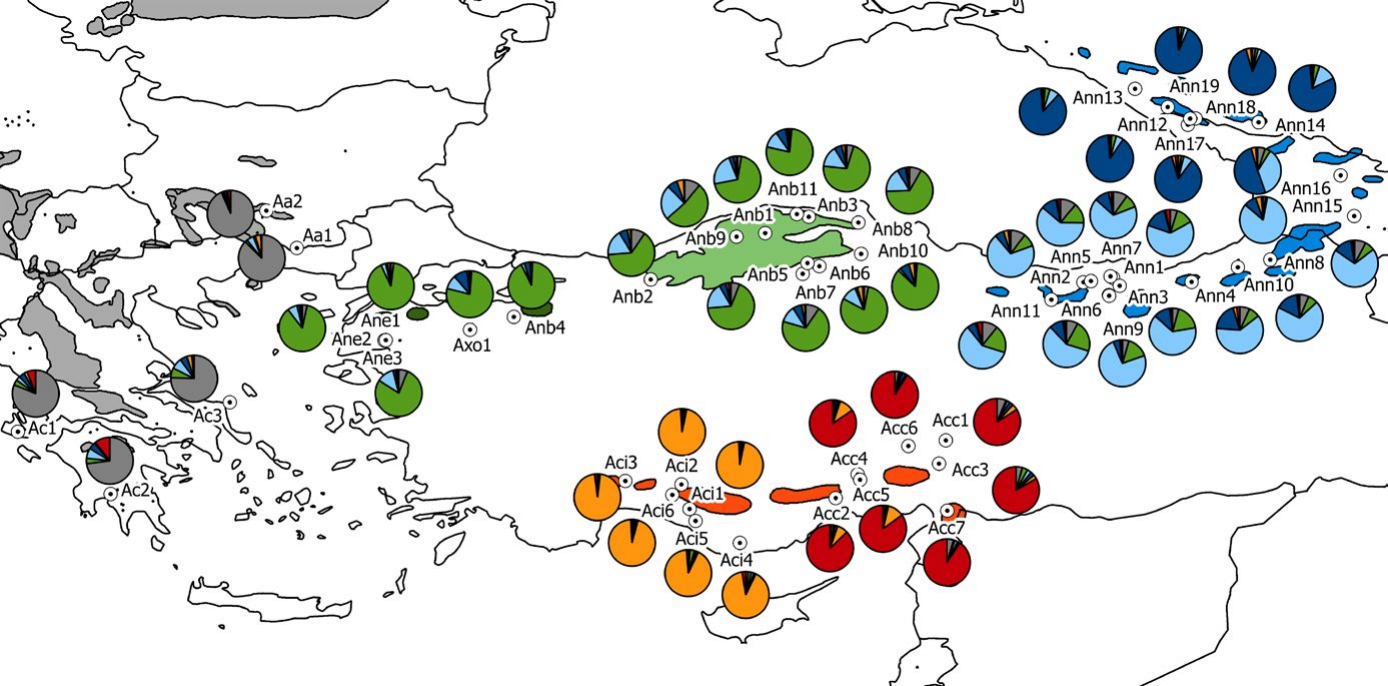
Results of the whole‐dataset structure analysis superimposed over the map of the eastern‐Mediterranean. Charts represent inferred membership proportions of the studied populations. Distribution ranges of individual taxa (www.euforgen.org/species) are displayed in colors corresponding to the predominant structure cluster

A total number of 1,529 individuals from 52 indigenous populations were sampled; mostly in managed, naturally regenerated stands, less frequently in national parks, and nature reserves (Table S1; 30 adult individuals per population in most cases, with a minimum distance of 50 m between trees). The populations belonged to eight taxonomic groups: *Abies alba* (Bulgaria; 2 populations), *A. cephalonica* (Greece; 3), *A. nordmanniana s.s*. (Turkey, Georgia and Russia; 19), *A. bornmuelleriana* (Turkey; 11), *A. equi‐trojani* (Turkey; 3), *A*. ×*olcayana* (Turkey; 1 population), *A. cilicica s.s*. (Turkey; 7), and *A. isaurica* (Turkey; 6). An overview of geographical positions of these populations is shown in Figure 2. Twigs with one‐year‐old needles were collected.

### 2.2 DNA extraction and genetic analysis

DNA extraction protocol according to Doyle and Doyle (1987) was used to extract DNA from approximately 50 mg of silica gel dried needles. Eleven nuclear microsatellite loci previously identified in various *Abies* species were used SF324, SF333, SFb04, SF1 (Cremer et al., 2006), ABF18 (Saito, Lian, Hogetsu, & Ide, 2005), NFF7, NFF3, NFH15, NFH3 (Hansen, Vendramin, Sebastiani, & Edwards, 2005), AB15 (Rasmussen, Andersen, Frauenfelder, & Kollmann, 2008), and AfSI16 (Josserand et al., 2006). Two multiplex and three singleplex reactions were done. The multiplex reactions were set up as 5 μl mixtures using Qiagen Multiplex PCR kit (*Qiagen 206145, Hilden, Germany*) with Q solution according to manufacturer's instructions and approximately 50–100 ng DNA. Concentrations of primers were 0.20 μmol/L NFF3, 0.10 μmol/L NFH15, 0.20 μmol/L NFH3, and 0.10 μmol/L Ab15 in multiplex A; 0.15 μmol/L AfSI16, 0.15 μmol/L SF1, 0.30 μmol/L SFb4, and 0.15 μmol/L NFF7 in multiplex B. The singleplex reactions for primers SF 324, SF333, and ABF18 were done in 5 μl mixtures containing 0.2 U of Hot FirePol DNA polymerase (*Solis BioDyne, Tartu, Estonia*), 1× PCR buffer, 3 mmol/L MgCl_2_, 0.2 mmol/L dNTP, 0.8 mol/L BSA, and 0.3 μmol/L of the corresponding primer. Amplification programs consisted of initial step at 94°C for 15 min., followed by 35 cycles of 30 s denaturation at 94°C, 90 s annealing at 58°C, and 90 s elongation at 72°C. A final elongation step was performed at 60°C for 20 min. 0.9 μl of multiplex A product with 0.1 μl of size standard GeneScan 500 LIZ (Applied Biosystems, Foster City, California) and 9 μl of formamide was loaded as a first batch for separation on capillary analyzer ABI 3130 (Applied Biosystems). Second batch consisted of the singleplex products (0.6 μl of each) together with 0.8 μl of multiplex B product, 0.1 μl of size standard, and 7.3 μl of formamide. genemapper 4.0 (Applied Biosystems) was used to analyze the raw data and produce genotypes.

### 2.3 Data treatment

The presence of null alleles and linkage disequilibria between loci were tested with micro‐checker 2.2.3 (Van Oosterhout, Hutchinson, Wills, & Shipley, 2004) and arlequin 3.5.2 (Excoffier & Lischer, 2010). Subsequently, the loci SFb04 and NFF7 were removed from further analyses. The locus SF324 was also discarded because it contains the same repeat segment as SF1.

To assess the patterns of gene diversity and allelic richness, expected heterozygosities of populations were calculated using arlequin and allelic richness and private allelic richness were calculated by the program hp‐rare 1.1 (Kalinowski, 2005). Rarefaction to 22 and 140 gene copies was used to obtain allelic richness of individual populations and pooled groups (taxa), respectively. The populations Ann13, Aci4, and Aci5 were omitted from allelic richness analysis of individual populations because of low sample sizes, the same applies to *A. alba* and *A. × olcayana* for the analysis on the taxon level. Differences among taxa were tested by nonparametric Kruskal–Wallis test both on the subspecies and the species level; *A. alba* and *A. cephalonica* were excluded from this test.

The populations were checked for recent reduction in population sizes using the program bottleneck v. 1.2.02 (Cornuet & Luikart, 1997), which relates the observed gene diversity with the heterozygosity predicted on the basis of the observed number of alleles under the assumption of mutation‐drift equilibrium (Maruyama & Fuerst, 1985). The distribution of the expected heterozygosity was obtained through simulating the coalescent process under two‐phase model with the proportion of stepwise mutations of 95% and a variance among multi‐step mutations of 12%, as recommended by Piry, Luikart, and Cornuet (1999) for microsatellite loci. The probability of heterozygosity excess was tested using the Wilcoxon test (Cornuet & Luikart, 1997). In addition, the mode‐shift approach applied in bottleneck v. 1.2.02, based on search for transient distortions of allele frequency distributions induced by a reduction in population size, was used.

Several approaches to assess genetic differentiation of the studied populations were used. First approach was the program structure 2.3 (Pritchard, Stephens, & Donnelly, 2000). It was run 16 times for each number of clusters ranging from 1 to 10 with 100,000 burn‐in iterations and another 1,000,000 iterations without prior information on population structure. To determine the optimal number of clusters, the structure harvester script was used (version A.2 July 2014; Earl, 2012). After assigning the populations to clusters inferred by structure, the process was repeated second time for each cluster individually in order to find an additional genetic structure within each cluster. The same settings were used to run Structure, except for number of populations which ranged from 1 to 6, and again the optimal number of clusters was identified by structure harvester.

As a second approach, the Discriminant Analysis of Principal Components (DAPC) was used (Jombart, Devillard, & Balloux, 2010), implemented in the R package adegenet 1.4.2 (Jombart, 2008). To more closely inspect the structure of the less distinct clusters, the most differentiated ones were gradually removed. In all the steps, a number of PCs corresponding to ~90% of the total variance were retained. Assignment of populations into groups followed the current taxonomic classification and geography.

Finally, neighbor‐net was constructed in the program splitstree 4.13.1 (Huson, 1998). It was based on pairwise *F*_ST_ (Weir & Cockerham, 1984) calculated by the R package diveRsity 1.9.89 (Keenan, McGinnity, Cross, Crozier, & Prodöhl, 2013).

Analysis of molecular variance (Excoffier, Smouse & Quattro, 1992) was carried out using the arlequin software separately for species and subspecies/taxa. In the latter case, the outcomes of the structure analysis were considered, and the northern and southern populations of *A. nordmanniana* s.s. were considered as separate groups. The significance of variance components attributed to species/subspecies, populations, and individuals was tested using 99,999 random permutations.

Isolation by distance was tested according to Rousset (1997), regressing *F*_ST_/(1–*F*_ST_) (Slatkin, 1995) against logarithm of distance as recommended for a two‐dimensional case. Significance of the relationship between genetic dissimilarity and distance was tested by Mantel test, the strength of IBD was quantified using a reduced major axis (RMA) regression, as applied in ibdws v. 3.23 (Jensen, Bohonak, & Kelley, 2005). Confidence intervals of regression slopes were derived from 30,000 bootstraps over independent population pairs. Geographical distances were calculated by the program Geographical Distance Matrix Generator (Ersts, 2016). The Mantel tests were also performed separately for *A. nordmanniana s.l*., *A. nordmanniana s.s*. (whole subspecies and southern and northern populations separately), *A. bornmuelleriana*,* A. cilicica s.l*., *A. cilicica s.s*., and *A. isaurica* populations. The presence of barriers against gene flow was tested using the Monmonier's maximum‐differences algorithm implemented in the barrier 2.2 software (Manni, Guérard, & Heyer, 2004), which is aimed at the identification of abrupt changes in genetic differences between pairs of populations in the geographical context, based on a network obtained by Delaunay triangulation. Again, Slatkin's linearized *F*_ST_ (Slatkin, 1995) was used as a distance measure. Bootstrap support for the identified barriers was derived from 999 random resamplings. barrier was run with a successively increasing number of groups (*K*), starting from 1, until new barriers with a bootstrap support of at least 50% were appearing.

Speciation scenarios were checked by applying Approximate Bayesian Computation analysis (ABC) for specific segments of the speciation sequence displayed in Figure 1. The tested scenarios are shown in Fig. S1. The computations were done in diyabc v. 2.05 (Cornuet et al., 2014). In all runs, 300,000 datasets were simulated for each scenario. A total of 1% of the simulated datasets most similar to the observed data were used to estimate the relative posterior. Simulations applied the Generalized Stepwise Mutation (GSM) model. As we have no information on the distribution of mutation rates of microsatellite loci in *Abies*, a mean of 2 × 10^−4^ was used in analogy with *Picea* (Tsuda et al., 2016). One locus, showing excessively high frequency of single nucleotide insertions/deletions (AB15), was omitted from the analyses.

For the recalculation of the number of generations into a time scale, a reasonable estimate of generation time needed to be chosen. In silver fir, isolated trees start flowering at 20 years of age and trees in a canopy at 60 years (Wolf, 2003), but this is for sure not the average age of offspring‐producing trees in a natural forest ecosystem. Natural forests are characterized by a mosaic of developmental stages with cyclically changing standing stock and canopy closure. At the local culmination of standing stock, the height structure is typically one‐layer with closed canopy, whereas the lack of light is strongly limiting for regeneration (in spite of abundant flowering and cone‐bearing). In natural forests of silver fir (commonly growing in mixture with European beech, Norway spruce, and noble hardwoods), this stage usually occurs between 150 and 250 years of age of dominant trees (Korpeľ, 1995), before first canopy gaps enable survival of seedlings. *A. nordmanniana s.l*. grows in similar communities with similar stand structures (Mayer & Aksoy, 1986). On the other hand, firs in Asia Minor and the Caucasus may have experienced periods in their evolutionary history, when they spread by colonizing open areas; in that case, the generation turnover time must have been shorter. To account for this uncertainty and to use the analogy with *A. alba*, we counted with the generation turnover age of 150 ± 50 years (as a conservative choice) for the conversion.

## 3 RESULTS

Table 1 summarizes genetic variation within the studied taxa. Of course, two populations of *A. alba* cannot be considered representative of the whole species but for the other species and subspecies, sample sizes are sufficient to provide a realistic view. Both at the population and taxon level, the subspecies or regional populations of *A. nordmanniana s.l*. exhibited generally higher allelic richness and expected heterozygosity compared with *A. cilicica s.l*., while the difference in private allelic richness was nonsignificant. Within *A. nordmanniana,* neither allelic richness nor gene diversity within populations varies substantially and there is not visible difference pattern between the stenoendemics and the more widespread taxa or regional groups. On the taxon level, however, the endemics *A. equi‐trojani* contains clearly less alleles and private alleles (13.03 and 0.15, respectively) than the other subspecies (13.78‐15.71 and 0.39‐0.60, respectively). Expected heterozygosity did not show any geographical trend, nor it was associated with the range size: Endemic and geographically proximate *A. equi‐trojani* and *A. *×*olcayana* exhibited contrasting values. In *A. cilicica*, the subspecies *A. cilicica s.s*. showed genetic variation values comparable to *A. nordmanniana* (*A*_[140]_ = 15.21, *P*_[140]_ = 0.66); in contrast, *A*. *isaurica* was distinctly less diverse, with lower allelic richness (*A*_[140]_ = 10.50) and private allelic richness (*P*_[140]_ = 0.12).

**Table 1:** Genetic variation characteristics of the studied Mediterranean fir taxa (mean ± standard deviation)

Taxon	Population level				
	*N*	*A*_[22]_	*P*_[22]_	*H*_e_	*F*_ST_
*A. alba*	31.0 ± 1.4	6.15 ± 1.26	0.02 ± 0.01	0.750 ± 0.063	0.0564
*A. cephalonica*	31.0 ± 1.0	7.10 ± 0.35	0.00 ± 0.00	0.751 ± 0.016	0.0446
*A. nordmanniana*	29.5 ± 2.5	7.63 ± 0.94	0.05 ± 0.05	0.743 ± 0.042	0.0359
*s.s*. (north)	26.6 ± 7.9	7.22 ± 0.41	0.05 ± 0.04	0.716 ± 0.049	0.0280
*s.s*. (south)	29.4 ± 3.7	7.98 ± 0.82	0.05 ± 0.05	0.751 ± 0.024	0.0267
subsp. *bornmuelleriana*	29.7 ± 0.5	7.62 ± 1.31	0.04 ± 0.05	0.748 ± 0.055	0.0313
subsp. *equi‐trojani*	30.0 ± 0.0	7.40 ± 0.08	0.02 ± 0.01	0.761 ± 0.008	−0.0027
× *olcayana*	34.0	7.09	0.11	0.722	—
*A. cilicica*	29.6 ± 0.7	6.16 ± 0.87	0.06 ± 0.06	0.666 ± 0.055	0.1371
subsp. *cilicica*	29.6 ± 0.8	6.68 ± 0.36	0.08 ± 0.06	0.693 ± 0.045	0.0378
subsp. *isaurica*	29.7 ± 0.5	5.24 ± 0.71	0.02 ± 0.02	0.636 ± 0.052	0.0562
*P* ^1^		0.0035	0.2113	0.0015	
*P* ^2^		0.0002	0.8226	<0.0001	

	Regional/taxon level			
	*N*	*A*_[140]_	*P*_[140]_	*H*_e_
*A. alba*	62	NA	NA	0.777
*A. cephalonica*	93	13.26	0.22	0.776
*A. nordmanniana*	990	17.31	2.44	0.795
*s.s*. (north)	186	13.78	0.39	0.755
*s.s*. (south)	353	15.98	0.52	0.769
subsp. *bornmuelleriana*	327	15.71	0.60	0.779
subsp. *equi‐trojani*	90	13.03	0.15	0.762
× *olcayana*	34	NA	NA	0.722
*A. cilicica*	385	15.04	1.65	0.792
subsp. *cilicica*	207	15.21	0.66	0.721
subsp. *isaurica*	178	10.50	0.12	0.686

*N*, number of sampled individuals; *H*_e_, expected heterozygosity; *A*[*x*], allelic richness after rarefaction to *x* gene copies; *P*[*x*], private allelic richness after rarefaction to *x* gene copies; *F*_ST_, coefficient of differentiation within taxon/region; *P* ^1^ and *P* ^2^, significance of the Kruskal–Wallis test of the differences among subspecies and among species, respectively; NA, not applied.

None of the populations exhibited a significant recent bottleneck, generally, heterozygosity deficiency was more prevalent than heterozygosity excess (Table S2). The mode‐shift approach yielded identical outcome: even though a common graphical display of allele frequency distributions for 52 populations is difficult and the resulting picture is a bit confused, it is apparent that the distributions did not deviate from the L‐shape typical for nonbottlenecked populations at mutation‐drift equilibrium (Fig. S2). However, differentiation patterns indicate a strong fragmentation in some species: in spite of a range size comparable to the other studied *Abies* species and subspecies, *A. cilicica* subsp. *isaurica* was substantially more differentiated (*F*_ST_ = 0.0562). In contrast, absence of differentiation within *A. equi‐trojani* (*F*_ST_ = −0.0027) indicates that the three studied samples were in fact drawn from the same population (Table 1).

Results of the structure analysis strongly suggested the existence of six clusters (Δ*K* = 565; while the second highest Δ*K* was 11.7 for *K *=* *4; Table S3, Fig. S3a). The first cluster comprised *A. alba* and *A. cephalonica*. Second cluster consisted of *A. equi‐trojani*,* A*. ×*olcayana,* and *A. bornmuelleriana* populations (i.e. Northwestern and Northern Turkey). While the first and the second clusters joined several taxa, the third and the fourth clusters split the *A. nordmanniana s.s*. populations into two groups: northern (located around the Greater Caucasus) and southern (the Ponthic Mountains and the Small Caucasus). The fifth and the sixth clusters very clearly distinguished *A. cilicica* subsp. *isaurica* and *A. cilicica* subsp. *cilicica* (Figure 2).

Nevertheless, the multimodality of the Δ*K* distribution indicated a potential substructure. Secondary structure analyses were thus performed for each of the inferred clusters separately (Table S3, Fig. S3a,b). This procedure split the first (*A. alba* and *A. cephalonica*) cluster into the respective species. The second cluster covering northwestern and northern Turkey was subdivided into *A. equi‐trojani* and the rest, while *A*. ×*olcayana* was not clearly differentiated from *A. bornmuelleriana*. In the northern *A. nordmanniana s.s*. (fourth) cluster, the analysis rendered the Georgian population (Ann16) as different from the rest. This population is located further from the rest of the cluster and near the border with the southern group; otherwise the two clusters in *A. nordmanniana s.s*. did not exhibit any clear geographical pattern. In *A. isaurica* (fifth) cluster, the population Aci3 that formed an individual subcluster is located further from the rest of populations. Again, the other two subclusters were not clearly differentiated. Finally, in *A. cilicica s.s*., the secondary analysis singled out the three eastern populations that showed an admixture from the *A. isaurica* cluster.

Analysis of molecular variance showed that there is highly significant variation both at the species and the subspecies level, even though the respective variance components both represent around 5% of the total variation (Table 2). As species are logically more heterogeneous than subspecies, the interpopulation component is higher in the former case (4.50% and 2.94% for species and subspecies, respectively).

**Table 2:** Analysis of molecular variance among Mediterranean fir taxa at the species and subspecies/taxon level

Source of variation	*df*	Variance component			*F*‐statistics	
		Abs.	%	*p*		
Subspecies/taxa	8	0.0451	5.25	<.0001	0.0525	*F*_CT_
Populations	43	0.0252	2.94	<.0001	0.0310	*F*_SC_
Within populations	3006	0.7888	91.81	<.0001		
Total	3057	0.8591	100		0.0819	*F*_ST_
Species	3	0.0473	5.40	<.0001	0.0540	*F*_CT_
Populations	48	0.0394	4.50	<.0001	0.0475	*F*_SC_
Within populations	3006	0.7888	90.10	<.0001		
Total	3057	0.8755	100		0.0990	*F*_ST_

**Figure 3:**
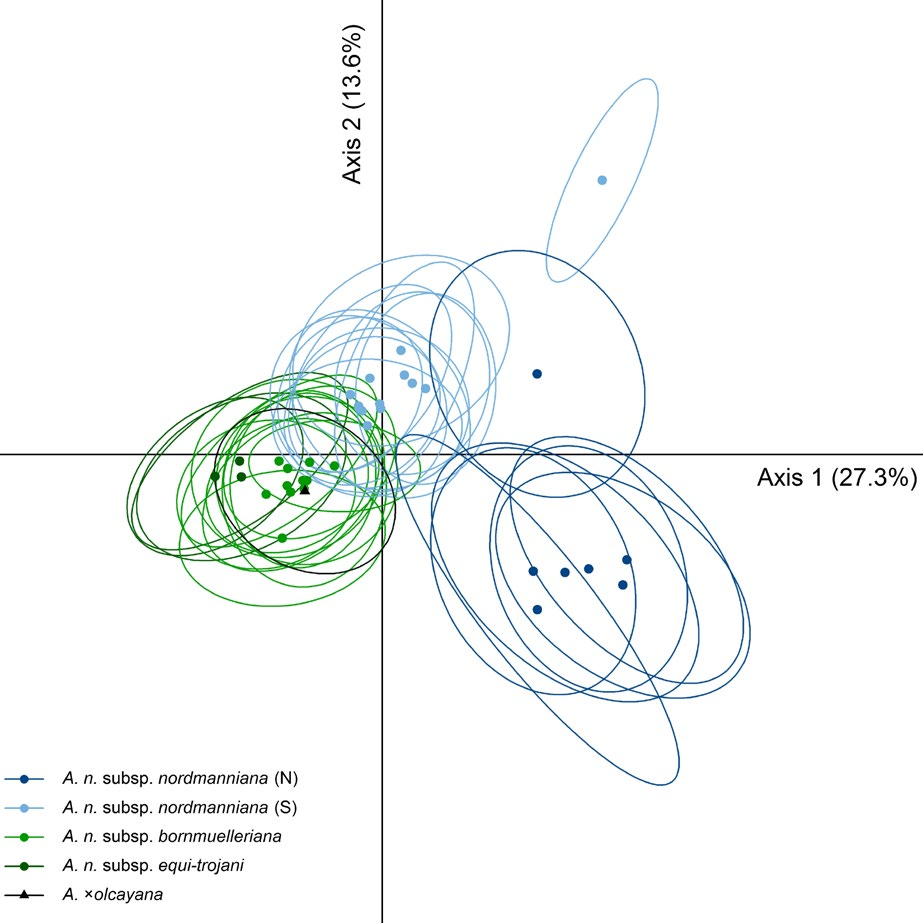
Discriminant analysis of principal components of the *Abies nordmanniana* populations. Only centroids and inertia ellipses are shown. Ann, *A. nordmanniana s.s*.; Anb, *A. nordmanniana* subsp. *bornmuelleriana*; Ane, *A. nordmanniana* subsp. *equi‐trojani*; Axo, *Abies* ×*olcayana*

Discriminant analysis of principal components showed a clear and strong differentiation of the two *A. cilicica* subspecies, both from the rest and from each other (Fig. S4). To study the structure of the *A. nordmanniana* populations, *A. cilicica*,* A. alba,* and *A. cephalonica* populations were removed from the analysis. The results displayed on Figure 3 differentiated all the subspecies with few exceptions: *A*. ×*olcayana* was grouped together with *A. bornmuelleriana* and the Georgian populations (Ann15 and Ann16) were positioned separately. The northern *A. nordmanniana s.s*. group was the most distinct, while the *A. equi‐trojani* was the least distinct group, close to *A. bornmuelleriana*.

**Figure 4:**
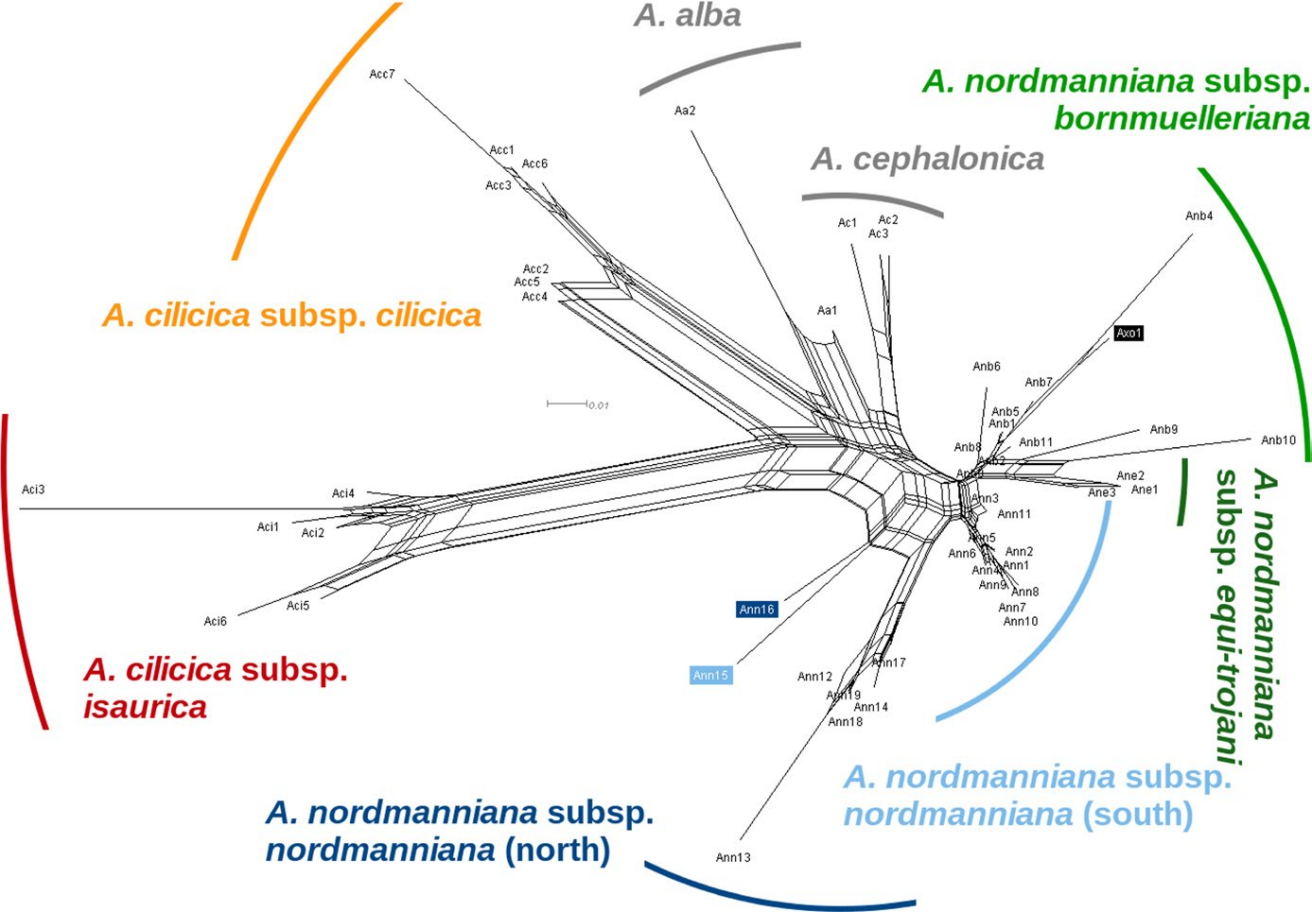
Neighbor‐net chart of the studied populations based on pairwise genetic distances (*F*_ST_; Weir & Cockerham, 1984)

The neighbor‐net (Figure 4) similarly showed that the two subspecies of *A. cilicica* were by far the most differentiated groups. They were followed by the *A. alba* and *A. cephalonica* populations. The *A. nordmanniana* groups were more mixed. The northern populations of *A. nordmanniana s.s*. represented the most distinct group in this cluster. However, it was possible to identify all the other groups except for *A*. ×*olcayana*.

Isolation by distance across the whole population set was significant (*p *<* *.0001, Mantel test), the RMA slope was 0.1685 ($95\%\text{CI}=〈0.1596,0.1773〉$) but RMA explained less than 5% of the total variation only (*R* ^2^ = 0.0478). Otherwise, a significant IBD was observed across *A. nordmanniana* s.l. (*p *=* *.0036) and in the southern group of *A. nordmanniana* s.s. (*p *=* *.0207) (Table S4).

The barrier analysis coincided well with both distance‐based and model‐based approaches. The Monmonier's algorithm yielded 10 barriers with a bootstrap support of >0.5 (Fig. S5), whereas for higher numbers of groups (*K *>* *10), no new barriers appeared. The strongest barriers appearing already at the lowest number of groups *K* were those separating *A. cilicica* from the remaining species and separating *A. cilicica* subspecies from each other. Moreover, well‐supported barriers appeared also among the populations of *A. isaurica*. Other barriers separated European species from the Asian, *A. equi‐trojani* from the rest of *A. nordmanniana* s.l., and the Caucasian *A. nordmanniana* s.s. from the populations of Asia Minor. The easternmost *A. bornmuelleriana* population (Samsun‐Vezirköprü) and both easternmost *A. nordmanniana* s.s. populations in Georgia (Ritsa 2 and Ritsa 3) also formed separate groups.

ABC simulations were run for a set of scenarios of phylogeny and past demographic changes in specific sets of fir eastern‐Mediterranean taxa, addressing the questions formulated in the Introduction section. In spite of a careful tuning of model priors, the performance of simulations was suboptimal (Table S5, Fig. S6). Estimates of effective population sizes (*N*_e_) and divergence times are shown in Table 3. For *A*. ×*olcayana,* the simulation gave a poor support for the hypothesis of being a taxon resulting from a past hybridization between *A. equi‐trojani* and *A. bornmuelleriana* but rather it is a clade belonging to the latter subspecies. The same is true for *A. equi‐trojani*: It represents an evolutionary branch of *A. bornmuelleriana*, which diverged from the parental line around 300–400 ky before present, rather than being a hybrid of *A. alba* and *A. bornmuelleriana*. The two alternative scenarios for *A. nordmanniana* s.l. had quite equivalent posterior probabilities (0.608 vs. 0.392) indicating that the intention of drawing a scenario for the whole species based on the available data was too ambitious. Here the simulations yielded earlier separation of *A. equi‐trojani* from *A. bornmuelleriana* (621 ky BP) and a very similar divergence time of the North‐Caucasian *A. nordmanniana* s.s. populations from the South‐Caucasian and Anatolian ones (692 ky BP). In *A. cilicica*, extreme differentiation and low allelic richness of *A. isaurica* suggest that this subspecies may have experienced a severe bottleneck in its history. However, the pure‐divergence model with stable effective population sizes in both subspecies (although lower in *A. isaurica*) performed better than the model including *N*_e_ change and indicated an early split of the subspecies (695 ky BP).

**Table 3:** Posterior estimates of the parameters of the demographic inference based on the Approximate Bayesian Computation for the best‐supported scenarios in different constellations of Mediterranean fir taxa or regional populations

Parameter	Mode	95% Confidence interval
*A. bornmuelleriana—A. equi trojani—A*. × *olcayana*		
Posterior probability^a^ of scenario 2	0.7547	0.7427–0.7666
*N*_e_ (Ane)	32,300	12,100–57,200
*N*_e_ (Anb)	63,200	28,700–93,800
*N*_e_ (Axo)	10,400	2,960–19,400
*t*_1_ (divergence Axo from Anb)	521^2^ (78 ± 26 ky^c^)	152–2,000^b^
*t*_2_ (divergence Ane from Anb)	2,790 (419 ± 139 ky)	931–8,720
*A. bornmuelleriana—A. equi trojani—A*. *alba*		
Posterior probability of scenario 2	0.9156	0.9089–0.9224
*N*_e_ (ancestor)	4,260	950–84,400
*N*_e_ (Ane)	26,200	9,470–57,300
*N*_e_ (Anb)	84,500	45,400–98,900
*N*_e_ (Aa)	25,800	8,620–75,900
*t*_1_ (divergence Ane from Anb)	2,010 (301 ± 101 ky)	490–7,650
*t*_2_ (divergence Aa and Anb from a common ancestor)	24,900 (3.73 ± 1.25My)	18,600–110,000
*A. nordmanniana s.l*.		
Posterior probability of scenario 1	0.6080	0.5945–6,215
*N*_e_ (ancestor)	1,690	1,180–35,100
*N*_e_ (Ane)	26,700	8,060–56,700
*N*_e_ (Anb)	72,500	35,500–97,000
*N*_e_ (AnnS)	82,400	48,300–98,200
*N*_e_ (AnnN)	37,900	13,500–93,400
*t*_1_ (divergence AnnN from AnnS)	4,610 (692 ± 231 ky)	924–9,390
*t*_2_ (divergence Ane from Anb)	4,140 (621 ± 207 ky)	723–9,220
*t*_3_ (divergence AnnS and Anb from a common ancestor	20,500 (3.08 ± 1.03 My)	7,510–58,700
*A. cilicica s.l*.		
Posterior probability of scenario 1	0.8247	0.8148–0.8345
*N*_e_ (Aci)	16,400	6,290–74,700
*N*_e_ (Acc)	83,200	26,000–98,200
*t*_1_ (divergence Aci from Acc)	4,630 (695 ± 232 ky)	1,070–61,200

*N*_e_, effective population size; *t*, time of divergence.

Aa, *A. alba*; Ane, *A. nordmanniana* subsp. *equi‐trojani*; Axo, *A*. ×*olcayana*; Anb, *A. nordmanniana* subsp. *bornmuelleriana*; Ann, *A. nordmanniana s.s*. (S and N for the southern and northern part, respectively); Aci, *A. cilicica* subsp. *isaurica*; Acc, *A. cilicica* subsp. *cilicica*.

a Posterior probabilities for the scenarios with the highest posterior probability of 10,000 sets of summary statistics most similar to the observed data through logistic regression.

b Generations.

c 1 generation = 150 ± 50 years.

## 4 DISCUSSION

### 4.1 Genetic variation

In contrast to continental Europe, the Mediterranean basin as a hotspot of species and genetic diversity (Cuttelod, García, Abdul Malak, Temple, & Katariya, 2008; Fady‐Welterlen, 2005) was not as profoundly affected by Pleistocene glacial cycles as the more northern areas, and many taxa have persisted since the Tertiary (Palamarev, 1989). The degree of range fragmentation seems to be the main determinant of diversity and differentiation patterns in eastern‐Mediterranean firs. Even though our study did not reveal any signs of a recent bottleneck (in accordance with Awad et al., 2014 but in contrast to Sękiewicz et al., 2015), population size and connectivity obviously affected genetic diversity levels. Geographical distribution of allelic variation largely corresponds with the observations of Scaltsoyiannes et al. (1999).

The highest allelic richness (both on the population and the taxon level) and lowest interpopulation differentiation were observed in *A. bornmuelleriana* and the Anatolian populations of *A. nordmanniana s.s*. (southern group). The ranges of these two taxa are not completely continuous but they form generally large local forest complexes (Alizoti, Fady, Prada, & Vendramin, 2011; Arbez, 1969; Mayer & Aksoy, 1986). The situation in the Caucasian part of the *A. nordmanniana s.s*. range (northern group) is not substantially different but the more rugged topography may contribute to isolation of local populations (Nakhutsrishvili, Zazanashvili, Batsatsashvili, & Montalvo Mancheno, 2015), and potentially to the loss of alleles by genetic drift, reflected in a lower allelic richness especially on the regional level. The northern group is also located on the margin of the distribution of Mediterranean firs as such, the potential for genetic enrichment by gene flow from related species is very limited, also because of restricted pollen dispersal in firs: most fir pollen is deposited within tens of meters from the source tree (compared to thousands of meters in the case of pine; Poska & Pidek, 2010).

The populations of the westernmost subspecies *A. equi‐trojani* and the putative hybrid *A*. ×*olcayana* are separated by a large gap from the rest of *A. nordmanniana s.l*. Moreover, the Kazdağı subpopulation suffers from logging, acid rain, and degradation of the habitat (Knees & Gardner, 2011), which all contribute to the loss of fertile trees and prevent that the permanently small population size increases. In spite of this, *A. equi‐trojani* retained relatively high allelic richness (at least at the population level) which, seen from the perspective of conservation, is a good news.

In accordance with the observation of Tayanç, Çengel, Kandemir, and Velioğlu (2012), levels of allelic richness and gene diversity in *A. cilicica* (notably in *A*. *isaurica*) were generally lower compared with the remaining taxa. The reasons may be associated with biogeography. The range of *A. cilicica* is more fragmented and the whole Taurus mountain range suffers from long‐term forest degradation, especially at lower elevations (Gardner & Knees, 2013; Mayer & Aksoy, 1986). Even though Sękiewicz et al. (2015) did not detect a difference in diversity between the two subspecies of *A. cilicica*, they reported a significant inbreeding and low effective population sizes in the studied populations.

Differences in biogeographical patterns are also reflected in the levels of differentiation. We observed a significant but very weak isolation by distance across all studied taxa. However, at the species/subspecies level, *A. bornmuelleriana* was the sole taxon where significant isolation by distance was observed, which may, however, be associated with small sample sizes (in terms of the numbers of populations) within most taxa. Along with a cline‐like pattern of structure group proportions, this indicates that a certain level of gene exchange between neighboring populations occurs in *Abies nordmanniana s.l*. The intrataxon differentiation level in Ponthic firs is clearly correlated with the range size: no differentiation in stenoendemic *A. equi‐trojani* vs. moderate levels in *A. bornmuelleriana* and regional populations of *A. nordmanniana s.s*. Unexpected was a strong differentiation between the North‐Turkish/Georgian and Russian populations of *A. nordmanniana s.s*. In contrast to our study, Hansen, Kjær et al. (2005) observed a low genetic differentiation between the *A. nordmanniana s.s*. populations from the Greater and Lesser Caucasus in chloroplast microsatellites.

In *A. cilicica*, subspecies are highly differentiated again, *A*. *isaurica* substantially more than *A*. *cilicica* s.s., and are considerably differentiated also to each other. Sękiewicz et al. (2015) also found a clear genetic differentiation between *A. cilicica* subspecies and attributed it to isolation by distance. Our observation did not confirm this, even though a lack of significant IBD patterns may result from small sample sizes per subspecies. Admixture between the two subspecies was negligible, limited to the closest populations, and asymmetric: the structure analysis revealed admixture of *A. isaurica* gene pool in the three westernmost *A*. *cilicica s.s*. populations. Awad et al. (2014) found similarly asymmetric migration between two demes of *A. cilicica s.s*. in Lebanon.

Our data provided a consistent geographical pattern, the detected genetic clusters are associated with mountain ranges: the western part of the Greater Caucasus and the Lesser Caucasus/Ponthic Mountains in the case of northern and southern group of *A. nordmanniana s.s*., respectively; Köroğlu, Ilgaz, and Küre Mountains in the case of *A. bornmuelleriana*; Kazdağı Mountains in the case of *A. equi‐trojani*; and the Western and Eastern Taurus in the case of *A. isaurica* and *A. cilicica s.s*., respectively. Several of these clusters were also well‐supported by the genetic barriers found, especially in the case of *A. cilicica* and its subspecies. However, significant barriers also appeared within subspecies and regional populations, indicating strong effects of range fragmentation; this was mainly the case of *A. isaurica* growing in a harsh climate of southern Turkey. Obviously, geographical barriers and range discontinuities associated with the Pleistocene climatic fluctuations effectively prevented gene flow, which would otherwise contribute to homogenization of genetic structures, at least at neutral loci.

### 4.2 Phylogeny

Even though the set of analyzed nSSR loci offers a quite poor coverage of the genome, the combination of distance‐based (neighbor‐net, DAPC) and model‐based Bayesian approaches (structure, ABC) allowed getting an insight into phylogeny and demographic processes in eastern‐Mediterranean firs.

All species of the section *Abies* are easily crossable with each other, as shown by artificial crossing experiments, which produced viable and mostly well‐growing and fertile hybrids (Greguss & Paule, 1988). This is even true for *A. cilicica*. There is thus potential for extensive interspecific gene flow and introgression; however, not much evidence. The structure analysis revealed certain (generally low) levels of admixture in all populations, exhibiting a geographical trend only across *A. nordmanniana s.l*. and partly across *A. cilicica s.l*., which may reflect recent gene flow. A relatively high proportion of *A. alba/cephalonica* gene pool in the southern *A. nordmanniana* populations is more plausibly due to shared ancestral polymorphisms, as this gene pool is mostly absent in geographically intermediate *A. bornmuelleriana*. At the same time, neither structure nor ABC simulations supported the hypotheses of hybrid origin of *A*. × *olcayana* and *A. equi‐trojani*, suggested by Ata and Merev (1987) and Klaehn and Winieski (1962). In both cases, the putative hybrid taxon was shown to represent a quite recent evolutionary branch of *A. bornmuelleriana*.

The ABC‐based estimates of divergence times and effective population sizes should be considered a crude approximation. The problem of a small number of loci was already mentioned, and tandem‐repeat loci are known to be more prone to homoplasy than the other types of markers. Moreover, we have no reliable information about mutation rates in nSSR loci in the genus *Abies* and relied on the study performed in another conifer genus (however, the mean mutation rate used by Tsuda et al., 2016 also relies on indirect evidence only). Second, the current version of the diyabc algorithms does not take into account migration or pollen‐mediated gene flow among taxa which counteracts differentiation; this may lead to underestimation of divergence times (van Loo, Hintsteiner, Pötzelsberger, Schüler, & Hasenauer, 2015; Semerikov, Semerikova, Polezhaeva, Kosintsev, & Lascoux, 2013). Finally, we did not include changes of effective population sizes in the evolutionary past of the taxa. Asia Minor was much less influenced by Pleistocene climatic fluctuations than most of Europe (Sarıkaya, Çiner, & Zreda, 2011). This does not exclude possible range contractions and expansions of fir species, which may have affected gene pools and differentiation patterns. However, we have no reliable information about these changes to suggest a fact‐based scenario, this issue was thus not considered in our simulations. The only exception was extremely differentiated *A. isaurica*.

In our simulations, many additional scenarios could be formulated but we tried to avoid those, which are unrealistic with regard to biogeography and fossil evidence or cannot be plausibly modeled with the available data. Although the outcomes of different simulations are generally quite consistent, several discrepancies appeared. The mode of divergence times is shorter for the split from the common ancestor of *A. alba* and *A. bornmuelleriana* than for *A. bornmuelleriana* and *A. nordmanniana*. This may be due to a small sample size in *A. alba*: only two geographically very proximate silver fir populations from the Balkans were included in the analysis, which do not represent the gene pool of the species as such. This is also reflected in a very broad confidence interval of the divergence time in this case. Moreover, as suggested by Linares (2011), gene flow between fir populations on both sides of the present Bospor strait was probably maintained during most of the Pleistocene.

For the interpretation of ABC outcomes in terms of a temporal scale, a correct estimation of the average generation turnover time is crucial. In long‐lived forest trees producing gametes for a large part of their lifespan, this is always associated with a big uncertainty, and true generation times may depend from species and ecological conditions: for example, for *A. cilicica* growing in forests with looser stand structure (Mayer & Aksoy, 1986), the average of 150 years may be an overestimation. If the proposed generation time is accepted, most speciation events occurred during the late Pliocene and Pleistocene. Thus, the estimated times of species or subspecies divergence fit well with the outline of the phylogeny of *Abies* in the eastern‐Mediterranean space as drawn by Linares (2011) and Palamarev (1989).

### 4.3 Implications for taxonomy and conservation

As already mentioned, the classification of *A. cilicica* has changed in the past, from section *Piceaster* to section *Abies*. However, these sections are both geographically and genetically close to each other anyway (Semerikova & Semerikov, 2014; Xiang et al., 2009, 2015). Still, the detected high differentiation of *A. cilicica* from the rest of the studied species is in line with the fact that *A. cilicica* is the least consistently classified into the section *Abies* and confirmed earlier findings of Scaltsoyiannes et al. (1999) and Bergmann et al. (2013). The taxonomical status of the three subspecies of *A. nordmanniana s.l*. is the most complicated issue in this study. Only *A. equi‐trojani* is recognized by Euro+Med PlantBase (Euro+Med, 2006) and IUCN (Knees & Gardner, 2011) and the genus‐level classifications usually deal only with *A. nordmanniana* as a whole species (Farjon & Rushforth, 1989; Liu, 1971; Xiang et al., 2009). On the other hand, many studies treated *A. equi‐trojani*,* A. bornmuelleriana,* and *A. nordmanniana s.s*. as separate species (Bergmann et al., 2013; Liepelt et al., 2010; Linares, 2011; Scaltsoyiannes et al., 1999). Contrary to the former classification, our findings clearly differentiated *A. bornmuelleriana* from the nominate subspecies. *A. equi‐trojani* was actually the group that was less clearly differentiated and tended to cluster under *A. bornmuelleriana*. Of course, *A. bornmuelleriana* is given as a synonym to *A. equi‐trojani* in the former classification, but the ranks given by the IUCN Red List do not agree with our results. Our findings support the classification given by Coode and Cullen (1965): *A. bornmuelleriana* and *A. equi‐trojani* are distinct groups, their differentiation is too low to support the classification as separate species. Nevertheless, from the point of view of conservation and forestry practice, it must be mentioned that all three taxa are treated as separate species in the OECD scheme for forest reproductive materials certification or in the European program for conservation of forest genetic resources Euforgen (Alizoti et al., 2011). There is not enough support for distinguishing *A. *×*olcayana* as a separate taxon (whether hybridogenous or not) as suggested by Ata and Merev (1987).

The studied species are generally classified as Least Concern in the IUCN Red List (http://www.iucnredlist.org), except *A. cilicica s.l*., which is Near Threatened as a whole, although local populations (mainly outside Turkey) are considered Critically Endangered. The outcomes of our study generally support this view. Even though genetic variation at neutral loci does not directly imply high adaptive variation, the observed relatively high levels of allelic richness and gene diversity within the studied taxa, including endemic and quite isolated *A. equi‐trojani*, are promising for their further survival. The only exception is *A. isaurica*; limited allelic richness and a strong differentiation from all remaining taxa make it a primary potential target of gene conservation efforts.

## CONFLICT OF INTERESTS

None declared.

## AUTHOR CONTRIBUTIONS

LP designed and organized the study, MH, DG, and DK made the analyses and statistical treatment, SK, HS, ITu, and ITv designed sampling and provided materials, MH and DG wrote the first draft, all authors contributed to the final text.
