# Supplementary material for: Genetic variation in Tertiary relics: The case of eastern‐Mediterranean Abies (Pinaceae)
Source: Ecol Evol. 2017 Oct 22;7(23):10018–30. doi: 10.1002/ece3.3519 (PMC5723589; doi:10.1002/ece3.3519)

Table S1 List of the studied populations of Mediterranean firs

Taxon	Population	Acronym	Latitude (°)	Longitude (°)	Altitude (m)	*N*	H_e	$A_{[22]}$	$P_{[22]}$	Status
Abies alba	Kirkovo	Aa1	41.286	25.372	480	30	0.754	6.530	0.010	MF
	Asenovgrad	Aa2	41.922	24.851	620	32	0.672	4.990	0.010	MF
Abies cephalonica	Kefalonia	Ac1	38.160	20.620	1200	30	0.741	6.520	0.000	MF
	Taygetos	Ac2	37.100	22.200	1270	32	0.696	6.520	0.000	MF
	Euboea	Ac3	38.660	24.230	960	31	0.715	6.970	0.000	MF
Abies nordmanniana subsp. *equi-trojani*	Kazdağları 1	Ane1	39.716	26.874	1500	30	0.731	6.910	0.010	NP
	Kazdağları 2	Ane2	39.715	26.862	1440	30	0.732	6.920	0.020	NP
	Kazdağları 3	Ane3	39.718	26.886	1560	30	0.736	6.930	0.020	NP
Abies nordmanniana ×olcayana	Yaylaçayırı	Axo1	39.896	28.322	770	34	0.676	6.590	0.100	NP
Abies nordmanniana subsp. *bornmuelleriana*	Kastamonu/Ballıdağ	Anb1	41.540	33.348	1520	30	0.735	7.890	0.010	MF
	Bolu/Bolu Dağı	Anb2	40.751	31.399	940	30	0.753	8.160	0.080	NP
	Kastamonu/Çatalzeytin	Anb3	41.817	34.090	1290	29	0.768	8.350	0.020	MF
	Bursa/Uludağ	Anb4	40.114	29.067	1580	30	0.644	5.580	0.000	NP
	Çankırı (Çankırı-Tosya)/Çankırı	Anb5	40.847	33.987	1710	29	0.717	7.140	0.050	MF
	Çorum/Iskilip	Anb6	40.977	34.270	1480	29	0.683	6.340	0.000	MF
	Kastamonu (Tosya Ilgazı)/Tosya	Anb7	41.031	34.068	1620	30	0.700	7.720	0.030	MF
	Sinop/Gerze	Anb8	41.720	34.935	1310	30	0.755	8.570	0.060	MF
	Karabük/Eflani	Anb9	41.479	32.859	1090	30	0.682	5.840	0.000	MF
	Samsun/Vezirköprü	Anb10	41.187	34.981	1370	30	0.626	5.430	0.010	MF
	Kastamonu/Inebolu	Anb11	41.864	33.887	1520	30	0.728	7.820	0.160	MF
Abies nordmanniana subsp. *nordmanniana*	Tonya/Trabzon	Ann1	40.779	39.273	1320	29	0.699	7.980	0.060	MF
	Espiye/Giresun	Ann2	40.704	38.768	1450	20	0.710	7.590	0.000	MF
	Zigana/Trabzon	Ann3	40.644	39.385	1840	30	0.752	7.970	0.060	MF
	İkizdere/Rize	Ann4	40.709	40.610	1720	28	0.691	7.640	0.030	MF
	Tirebolu Olucak Yaylası/Giresun	Ann5	40.724	38.897	1660	30	0.725	7.990	0.010	MF
	Torul Yıldız Köyü/Gümüşhane	Ann6	40.486	39.201	1860	30	0.723	7.960	0.020	MF
	Şalpazarı Sinlice Mevkii/Trabzon	Ann7	40.804	39.228	1450	30	0.652	7.500	0.030	MF
	Ardanuç/Artvin	Ann8	41.088	41.951	1900	30	0.686	7.010	0.140	MF
	Güvenli Köyü/Gümüşhane	Ann9	40.474	39.206	1775	30	0.719	7.540	0.080	MF
	Yusufeli/Artvin	Ann10	40.959	41.398	1700	30	0.683	6.880	0.000	MF
	Şebinkarahisar/Giresun	Ann11	40.408	38.222	1945	28	0.710	7.880	0.010	MF
	Krasnaja Polana	Ann12	43.686	40.205	600	31	0.652	6.490	0.040	NR
	Krasnodarsk	Ann13	43.995	39.647	950	9	0.556	-	-	NP
	Kaukaz	Ann14	43.426	41.752	1570	30	0.645	6.060	0.050	NR
	Borjomi	Ann15	41.830	43.380	1050	37	0.631	5.390	0.090	NP
	Ambrolauri	Ann16	42.523	43.149	1120	26	0.724	7.010	0.110	MF

	Ritsa1	Ann17	43.384	40.546	1290	30	0.678	7.190	0.080	NR
	Ritsa2	Ann18	43.492	40.678	1890	30	0.653	6.570	0.020	NR
	Ritsa3	Ann19	43.489	40.585	1240	30	0.650	6.480	0.000	NR
Abies cilicica subsp. *cilicica*	Göksun/K.Maraş	Acc1	38.013	36.424	1550	30	0.620	6.690	0.030	MF
	Gözne Karatepe/Mersin	Acc2	37.031	34.548	1320	30	0.740	6.410	0.050	MF
	Andırın/K.Maraş	Acc3	37.617	36.308	1895	28	0.660	6.300	0.060	MF
	Pozantı Armutlu/Adana	Acc4	37.434	34.924	1450	30	0.712	6.450	0.090	MF
	Karaisali Damlama /Adana	Acc5	37.343	34.964	1300	30	0.697	6.010	0.170	MF
	Bahçecik Bölgesi/Adana	Acc6	37.917	35.786	1390	30	0.668	6.470	0.000	MF
	Hassa Dedemli Yaylası/Hatay	Acc7	36.817	36.450	1546	29	0.601	5.820	0.080	MF
Abies cilicica subsp. *isaurica*	Gündoğmuş/Antalya	Aci1	36.846	32.060	1440	30	0.650	5.460	0.040	MF
	Bozkır Sapağı/Antalya	Aci2	37.263	31.921	1600	29	0.644	5.250	0.000	MF
	Sütçüler İşl. Müd. /Isparta	Aci3	37.322	30.966	1350	30	0.531	3.990	0.070	MF
	Abanoz Yaylası/Antalya	Aci4	36.267	32.917	1684	30	0.649	-	-	MF
	Alanya-Çukur/Antalya	Aci5	36.641	32.162	1025	29	0.679	-	-	MF
	Akseki-Freklibel/Antalya	Aci6	37.085	31.766	1314	30	0.620	5.230	0.020	MF

N – sample size, H_e – expected heterozygosity, $A_{[22]}$ – allelic richness after rarefaction to 22 gene copies, $P_{[22]}$ – private allelic richness after rarefaction to 22 gene copies

NR - nature reserve
NP - national park
MF - managed forest

Table S2 Deviations of heterozygosity from the mutation-drift equilibrium value and results of the Wilcoxon text

[1]probability of heterozygosity excess

Taxon	population	P^{1}	dH/SD
Abies alba	Aa1	0.3711	0.0634
	Aa2	0.4219	-0.0325
Abies cephalonica	Ac1	0.8750	-0.5225
	Ac2	0.9805	-0.7298
	Ac3	0.8086	-0.8876
Abies nordmanniana subsp. *equi-trojani*	Ane1	0.9727	-0.8324
	Ane2	0.9805	-0.6470
	Ane3	0.4727	-0.1139
Abies x *olcayana*	Axo1	0.9863	-1.0496
Abies nordmanniana subsp. *bornmuelleriana*	Anb1	0.9023	-1.3129
	Anb2	0.8438	-0.8938
	Anb3	0.7695	-0.4785
	Anb4	0.8086	-1.0030
	Anb5	0.8438	-0.8873
	Anb6	0.8750	-0.8724
	Anb7	0.9941	-1.5765
	Anb8	0.7266	-0.5931
	Anb9	0.3203	0.1438
	Anb10	0.9863	-1.2636
	Anb11	0.7266	-0.4774
Abies nordmanniana subsp. *nordmanniana*	Ann1	0.9961	-1.2186
	Ann2	0.8438	-0.3525
	Ann3	1.0000	-1.1764
	Ann4	0.9961	-1.1526
	Ann5	0.9961	-1.3580
	Ann6	0.9981	-1.2783
	Ann7	0.9961	-1.9609
	Ann8	1.0000	-1.4005
	Ann9	0.9981	-1.5136
	Ann10	1.0000	-1.2471
	Ann11	0.9023	-1.1531
	Ann12	0.7266	-0.6009
	Ann13	0.8516	-0.2430
	Ann14	0.8750	-0.6501
	Ann15	0.5273	-0.0549
	Ann16	0.8438	-0.6929
	Ann17	0.8438	-1.1318
	Ann18	0.9863	-0.9199
	Ann19	0.9023	-0.5533
Abies cilicica subsp. *cilicica*	Acc1	0.9941	-2.6676
	Acc2	0.7695	-0.1541
	Acc3	0.9863	-1.4618
	Acc4	0.9902	-0.9535
	Acc5	0.5273	-0.0839
	Acc6	0.9902	-1.2086

	Acc7	0.9727	-1.1908
Abies cilicica subsp. *isaurica*	Aci1	0.7695	-0.1948
	Aci2	0.5781	0.0289
	Aci3	0.9727	-1.3801
	Aci4	0.8438	-0.3853
	Aci5	0.3203	-0.3214
	Aci6	0.9863	-0.8818

Table S3 Table of Δ*K* values for all the performed Structure analyses.
cluster 1 – *A. alba* and *A. cephalonica* ; cluster 2 – *A. nordmanniana* except for subsp. *nordmanniana* ; cluster 3 – southern *A. nordmanniana* s.s. populations; cluster 4 – northern *A. nordmanniana* s.s. populations; cluster 5 – *A. cilicica* subsp. *isaurica* ; cluster 6 – *A. cilicica* subsp. *cilicica* . Bold-faced Δ*K* values denote the number of clusters used for the analyses.

Dataset	max *K*	*K* =2	*K* =3	*K* =4	*K* =5	*K* =6	*K* =7	*K* =8	*K* =9
All data	10	2.66	15.30	0.02	89.32	**119.71**	2.28	3.16	1.00
Cluster 1	6	**46.43**	0.51	14.95	0.18				
Cluster 2	6	**58.83**	34.65	2.62	4.15				
Cluster 3	6	**1.96**	0.77	1.64	1.47				
Cluster 4	6	11.97	**22.59**	2.21	3.57				
Cluster 5	6	30.18	**46.33**	2.93	3.12				
Cluster 6	6	**129.35**	3.20	0.65	1.88				

Table S4 Results of the isolation-by-distance analysis

Taxon	Mantel test				RMA regression					
	Z	*r*	*P* (H_0: *r* ≥0)	*P* (H_0: *r* ≤0)	intercept	95% CI	slope	95% CI	R^2	*n*
**all taxa**	305.4171	0.2185	>0.9999	**<0.0001**	-0.3715	(-0.3957, -0.3474)	0.1685	(0.1596, 0.1773)	0.0478	1326
A. cilicica	9.0200	0.0384	0.6712	0.3288	-0.2438	(-0.3120, -0.1755)	0.1312	(0.1012, 0.1612)	0.0015	78
A. cilicica subsp. *cilicica*	0.6869	0.2218	0.8299	0.1701	-0.0657	(-0.1044, -0.0270)	0.0413	(0.0220, 0.0606)	0.0492	21
A. cilicica subsp. *isaurica*	2.1950	-0.3473	0.1715	0.8285	0.4910	(0.2582, 0.7238)	-0.2186	(-0.3414, -0.0958)	0.1210	15
***A. nordmanniana* s.l.**	128.5166	0.1897	0.9964	**0.0036**	-0.3147	(-0.3479, -0.2814)	0.1572	(0.1404, 0.1740)	0.0360	561
A. nordmanniana subsp. *bornmuelleriana*	3.5159	-0.1059	0.3819	0.6181	0.1764	(0.1358, 0.2169)	-0.0690	(-0.0878, -0.0500)	0.0112	55
A. nordmanniana subsp. *nordmanniana*	50.6610	0.1875	0.9280	0.0720	-0.3074	(-0.3632, -0.2517)	0.1726	(0.1494, 0.1958)	0.0352	210
A.n.nordmanniana north	4.4474	-0.2528	0.2683	0.7317	0.5477	(0.3421, 0.7534)	-0.2252	(-0.3298, -0.1206)	0.0639	21
A.n.nordmanniana south	9.3719	0.4301	0.9793	**0.0207**	-0.1800	(-0.2371, -0.1230)	0.1240	(0.0960, 0.1520)	0.1850	66

Table S5 Model checking results for the tested scenarios

A. bornmuelleriana - A. equi-trojani - A. × olcayana		
summary statistics	observed value	proportion (simulated<observed)
NAL_1_1	14.1429	0.3475
NAL_1_2	10.1429	0.2005
NAL_1_3	19.7143	0.4765
HET_1_1	0.7784	0.058
HET_1_2	0.721	0.018 (*)
HET_1_3	0.7672	0.019 (*)
VAR_1_1	34.892	0.557
VAR_1_2	28.6841	0.417
VAR_1_3	28.806	0.381
N2P_1_1&2	16	0.296
N2P_1_1&3	20.5714	0.397
N2P_1_2&3	20	0.4665
H2P_1_1&2	0.7805	0.0655
H2P_1_1&3	0.7781	0.0195 (*)
H2P_1_2&3	0.7663	0.019 (*)
V2P_1_1&2	34.5601	0.538
V2P_1_1&3	31.5275	0.44
V2P_1_2&3	28.8728	0.38
FST_1_1&2	0.0571	0.788
FST_1_1&3	0.0319	0.568
FST_1_2&3	0.0238	0.7325
DAS_1_1&2	0.2059	0.988 (*)
DAS_1_1&3	0.1989	0.9845 (*)
DAS_1_2&3	0.2379	0.992 (**)

A. bornmuelleriana - A. equi-trojani- A. alba		
summary statistics	observed value	proportion (simulated<observed)
NAL_1_1	10.1429	0.2955
NAL_1_2	14.1429	0.4325
NAL_1_3	19.7143	0.5175
HET_1_1	0.7707	0.2850
HET_1_2	0.7784	0.0805
HET_1_3	0.7672	0.0670
VAR_1_1	13.7936	0.3690
VAR_1_2	34.8920	0.7450
VAR_1_3	28.8060	0.6290
N2P_1_1&2	16.7143	0.3645
N2P_1_1&3	20.5714	0.4450
N2P_1_2&3	20.5714	0.4890
H2P_1_1&2	0.8150	0.1040
H2P_1_1&3	0.7889	0.0390 (*)
H2P_1_2&3	0.7781	0.0400 (*)
V2P_1_1&2	40.8527	0.7890
V2P_1_1&3	30.1135	0.6280

V2P_1_2&3	31.5275	0.6760
FST_1_1&2	0.0983	0.6530
FST_1_1&3	0.0901	0.6980
FST_1_2&3	0.0319	0.7645
DAS_1_1&2	0.1459	0.9000
DAS_1_1&3	0.1586	0.9365
DAS_1_2&3	0.1989	0.9490
AML_1_2&1&3	0.0710	0.4955

***A. nordmanniana* s.l.**

summary statistics	observed value	proportion (simulated<observed)
NAL_1_1	14.1429	0.2595
NAL_1_2	20	0.3245
NAL_1_3	19.8571	0.286
NAL_1_4	16.4286	0.227
HET_1_1	0.7784	0.064
HET_1_2	0.7663	0.032 (*)
HET_1_3	0.7515	0.063
HET_1_4	0.7366	0.0245 (*)
VAR_1_1	34.892	0.714
VAR_1_2	28.8728	0.531
VAR_1_3	19.0109	0.273
VAR_1_4	16.4061	0.229
N2P_1_1&2	20.7143	0.2925
N2P_1_1&3	21	0.2565
N2P_1_1&4	19.8571	0.286
N2P_1_2&3	22.2857	0.253
N2P_1_2&4	22.2857	0.3115
N2P_1_3&4	21.1429	0.2695
H2P_1_1&2	0.7768	0.054
H2P_1_1&3	0.7705	0.025 (*)
H2P_1_1&4	0.7809	0.08
H2P_1_2&3	0.7735	0.024 (*)
H2P_1_2&4	0.776	0.026 (*)
H2P_1_3&4	0.7624	0.056
V2P_1_1&2	31.3702	0.577
V2P_1_1&3	29.1982	0.512
V2P_1_1&4	36.9071	0.685
V2P_1_2&3	27.7495	0.468
V2P_1_2&4	31.0561	0.549
V2P_1_3&4	19.1419	0.275
FST_1_1&2	0.0323	0.703
FST_1_1&3	0.0491	0.7645
FST_1_1&4	0.084	0.927
FST_1_2&3	0.0362	0.807
FST_1_2&4	0.0534	0.9025
FST_1_3&4	0.0439	0.944
DAS_1_1&2	0.1996	0.959 (*)
DAS_1_1&3	0.1974	0.974 (*)

DAS_1_1&4	0.1762	0.9605 (*)
DAS_1_2&3	0.2102	0.978 (*)
DAS_1_2&4	0.2055	0.9755 (*)
DAS_1_3&4	0.2229	0.973 (*)

A. cilicica

summary statistics	observed value	proportion (simulated<observed)
NAL_1_1	13.0000	0.2830
NAL_1_2	18.7143	0.4830
HET_1_1	0.7564	0.0410 (*)
VAR_1_1	26.0854	0.5170
VAR_1_2	42.5718	0.7940
N2P_1_1&2	19.8571	0.3815
V2P_1_1&2	39.7058	0.7270
DAS_1_1&2	0.1225	0.8405

Figure S1 Schemes of evolutionary scenarios tested in eastern Mediterranean *Abies* species

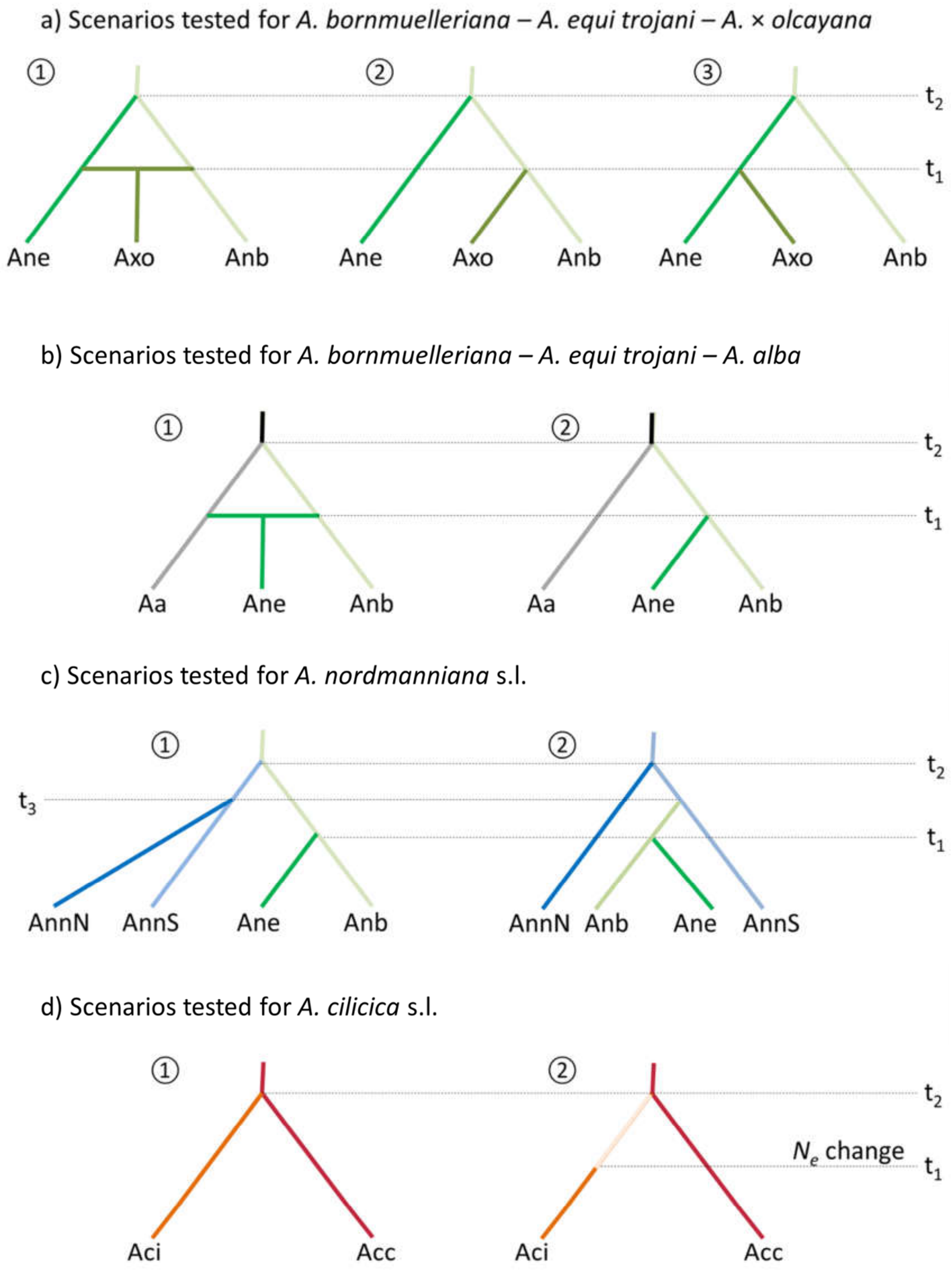

Figure S2 Distribution of allelic frequencies (mode-shift analysis of population bottlenecks) across all studied populations

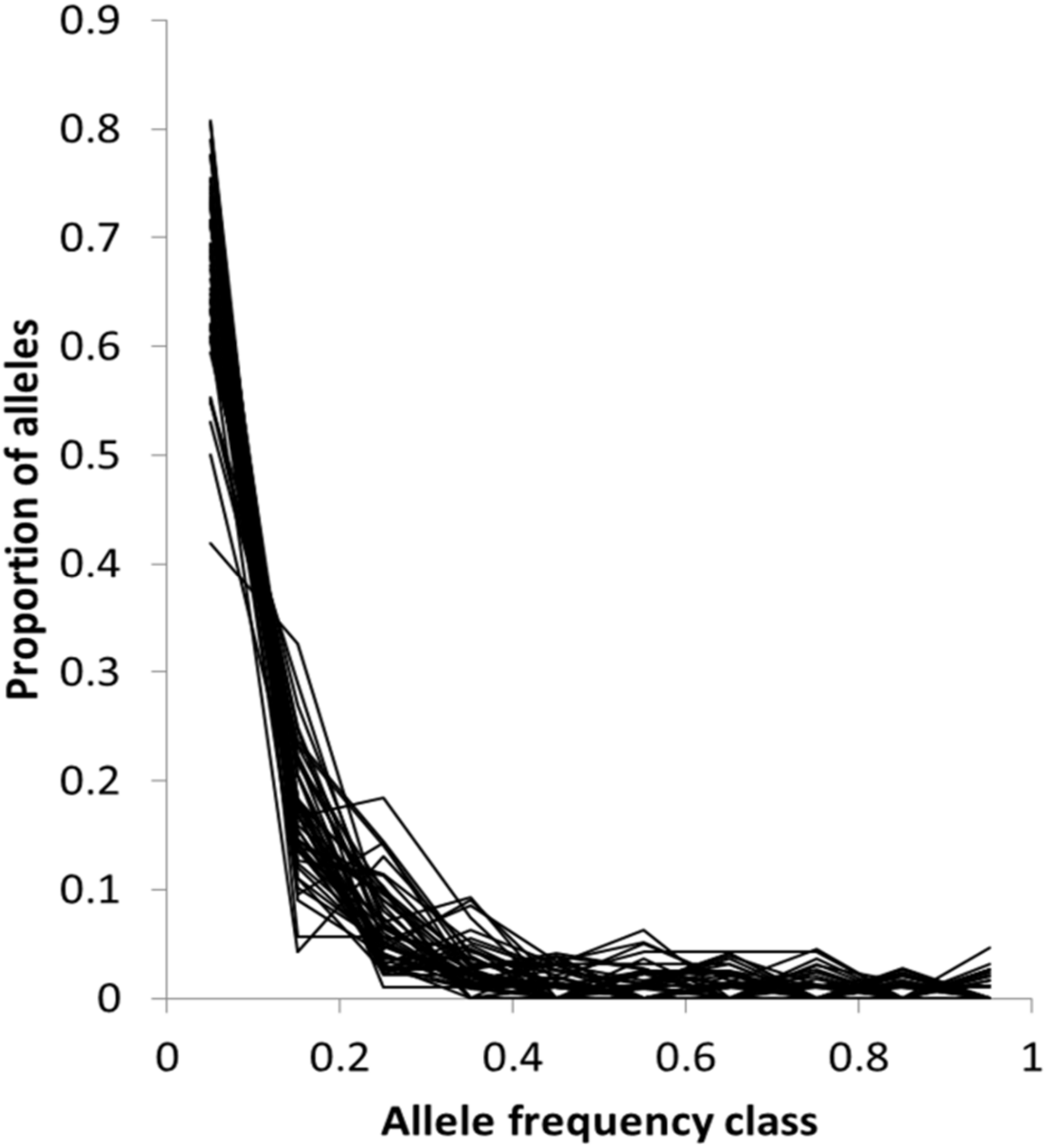

Figure S3a Determination of the optimum number of clusters in the Structure analyses using the method of Evanno et al. (2005).
Thin black line – mean ln P(D)|K (bars represent standard deviations), thick grey line – Δ*K* for different number of clusters *K*.

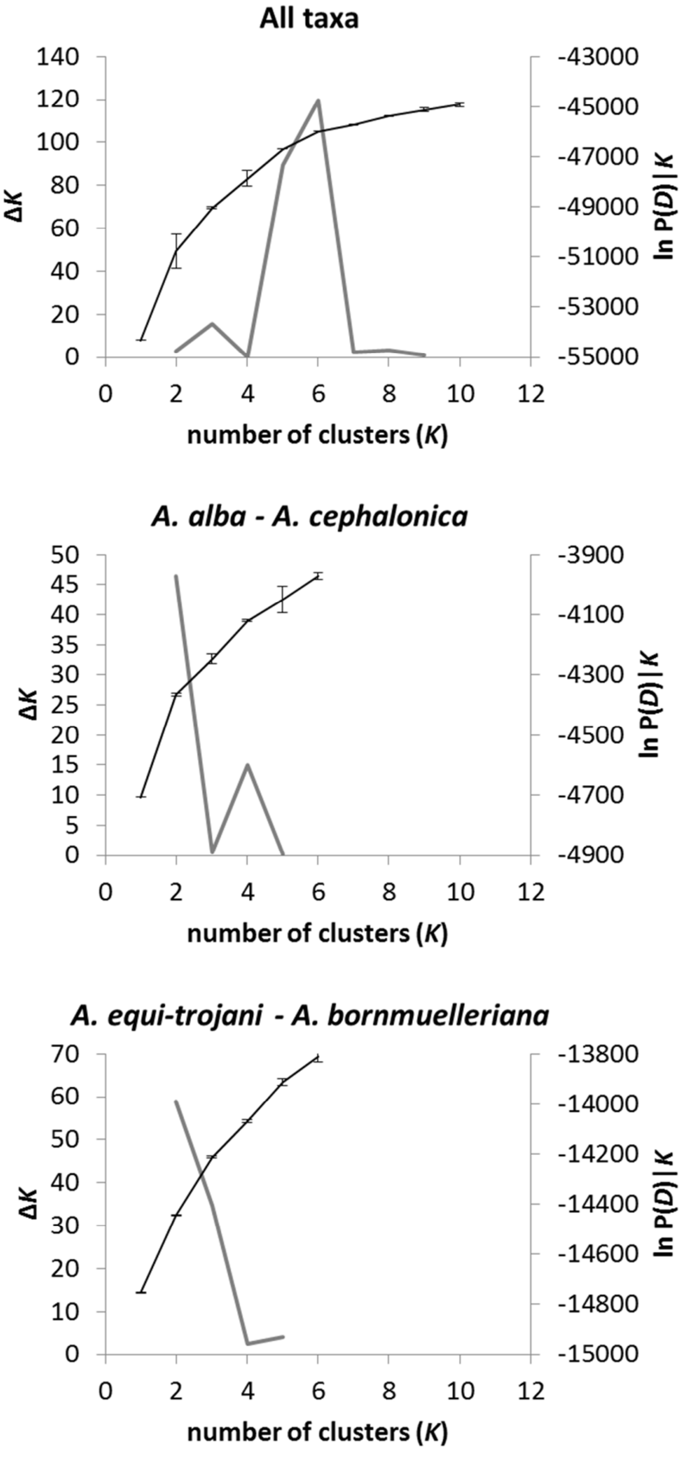

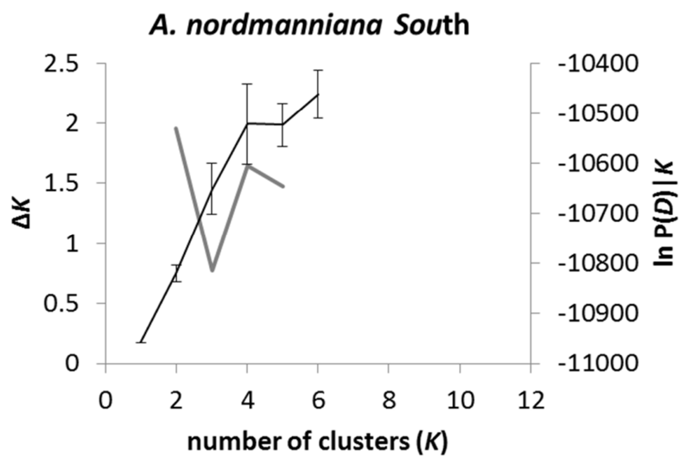
A. nordmanniana South
ΔK
ln P(D)|K
number of clusters (K)
2.5
2
1.5
1
0.5
0
-10400
-10500
-10600
-10700
-10800
-10900
-11000
0
2
4
6
8
10
12

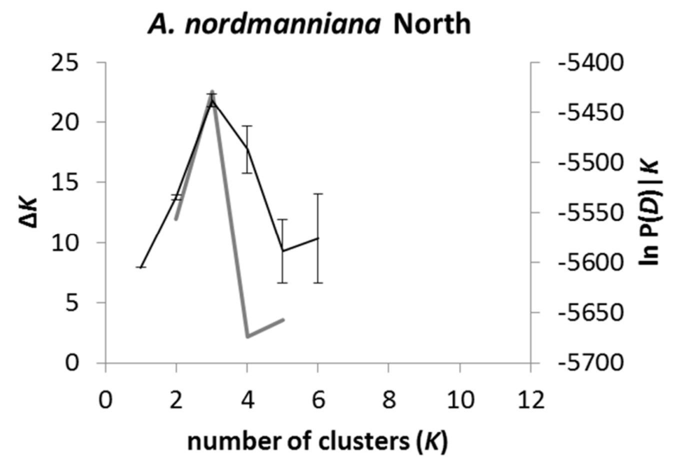
A. nordmanniana North
ΔK
ln P(D)|K
number of clusters (K)
25
20
15
10
5
0
-5400
-5450
-5500
-5550
-5600
-5650
-5700
0
2
4
6
8
10
12

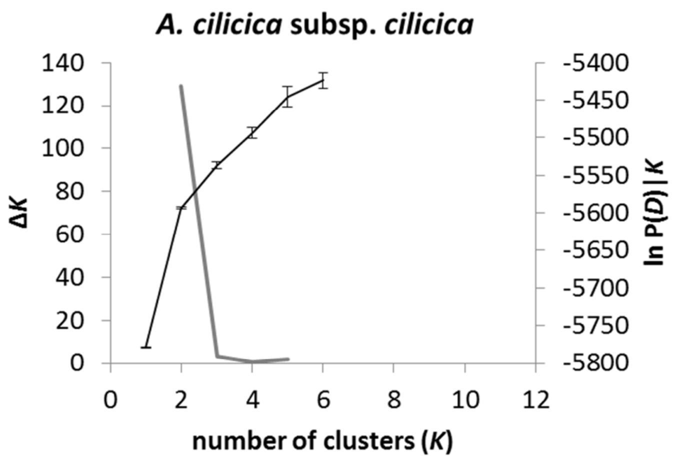
A. cilicica subsp. cilicica
ΔK
ln P(D)|K
number of clusters (K)
140
120
100
80
60
40
20
0
-5400
-5450
-5500
-5550
-5600
-5650
-5700
-5750
-5800
0
2
4
6
8
10
12

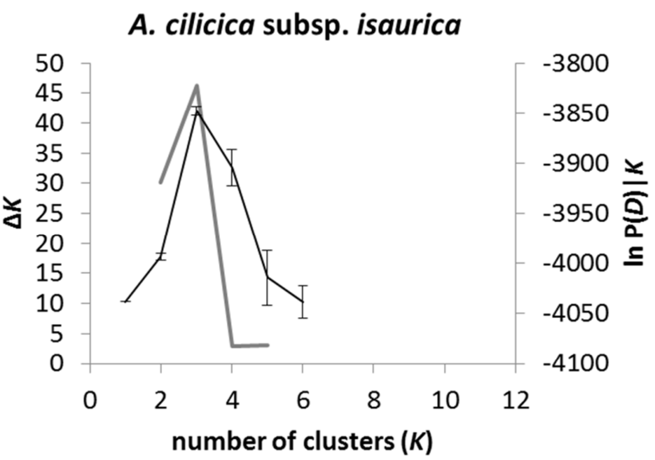

Reference:
Evanno G, Regnaut S, Goudet J (2005) Detecting the number of clusters of individuals using the software STRUCTURE: a simulation study. Mol Ecol 14:2611–2620

Figure S3b Results for both levels of the STRUCTURE analyses. Each column represents an individual tree sample. The populations are ordered by their geographic positions from east to west (or south to north in the case of AnnN). Upper row: initial analysis of the whole dataset; *K* =6. Lower row: secondary analyses of data partitioned into 6 population subsets based on the initial analysis. Colour of the predominant group in the analysis of the whole population set is used for the choice of colour shades in separate analysis by clusters (see also Fig. S4). Ace – *Abies cephalonica* ; Aa – *A. alba*; Ane – *A. nordmanniana* subsp. *equi-trojani*; Axo – *A. ×olcayana* ; Anb – *A. nordmanniana* subsp. *bornmuelleriana*; Ann – *A. nordmanniana* s.s. (S and N for the southern and northern part, respectively); Aci – *A. cilicica* subsp. *isaurica*; Acc – *A. cilicica* subsp. *cilicica*. Population numbering corresponds to Table S1.

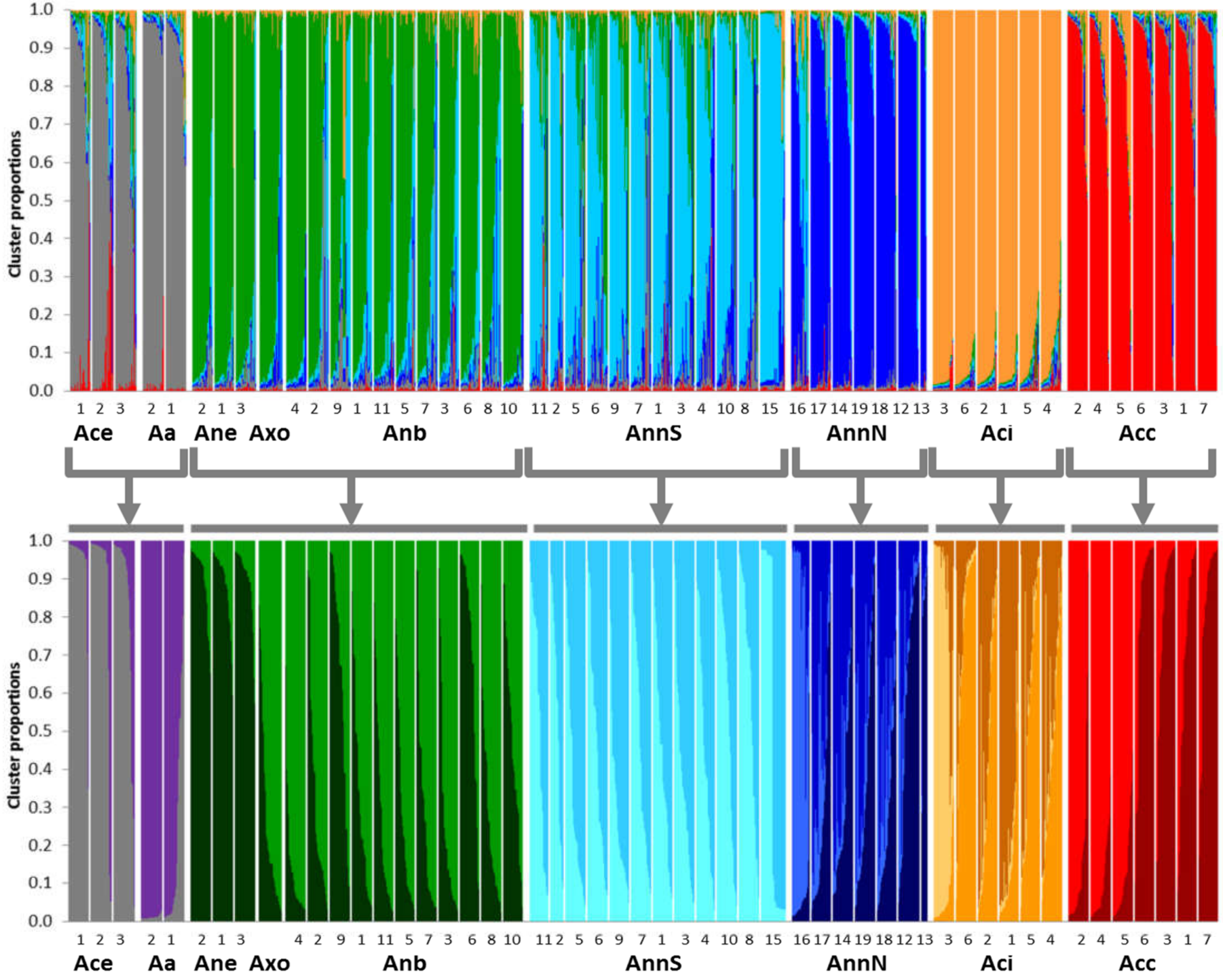

Figure S4 DAPC of all studied populations. Only centroids and inertia ellipses are shown.

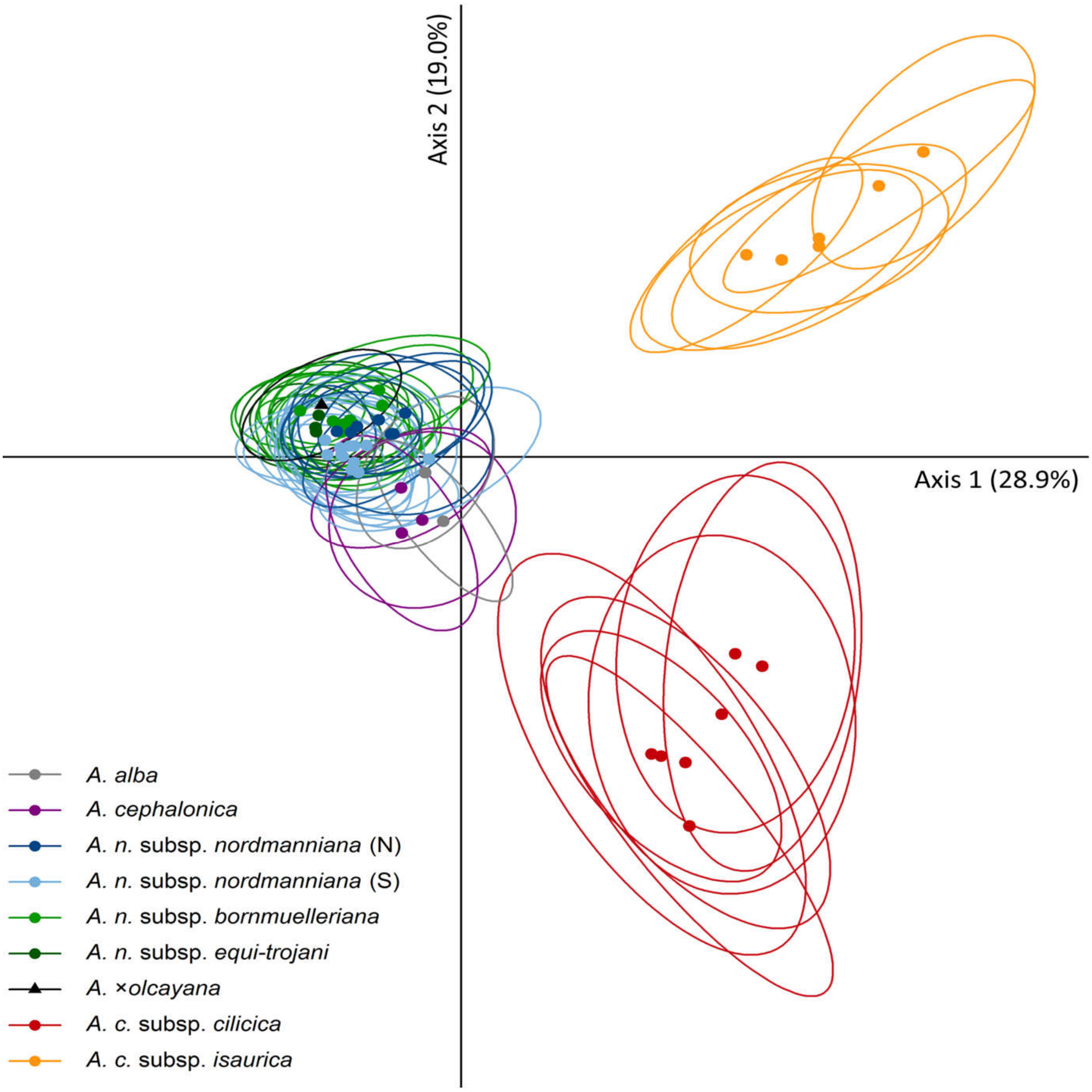

Figure S5 Genetic barriers among the populations of the studied Mediterranean fir taxa identified by the BARRIER analysis. Line thickness corresponds with the bootstrap support of each barrier. Only barriers with bootstrap support over 50% are displayed.

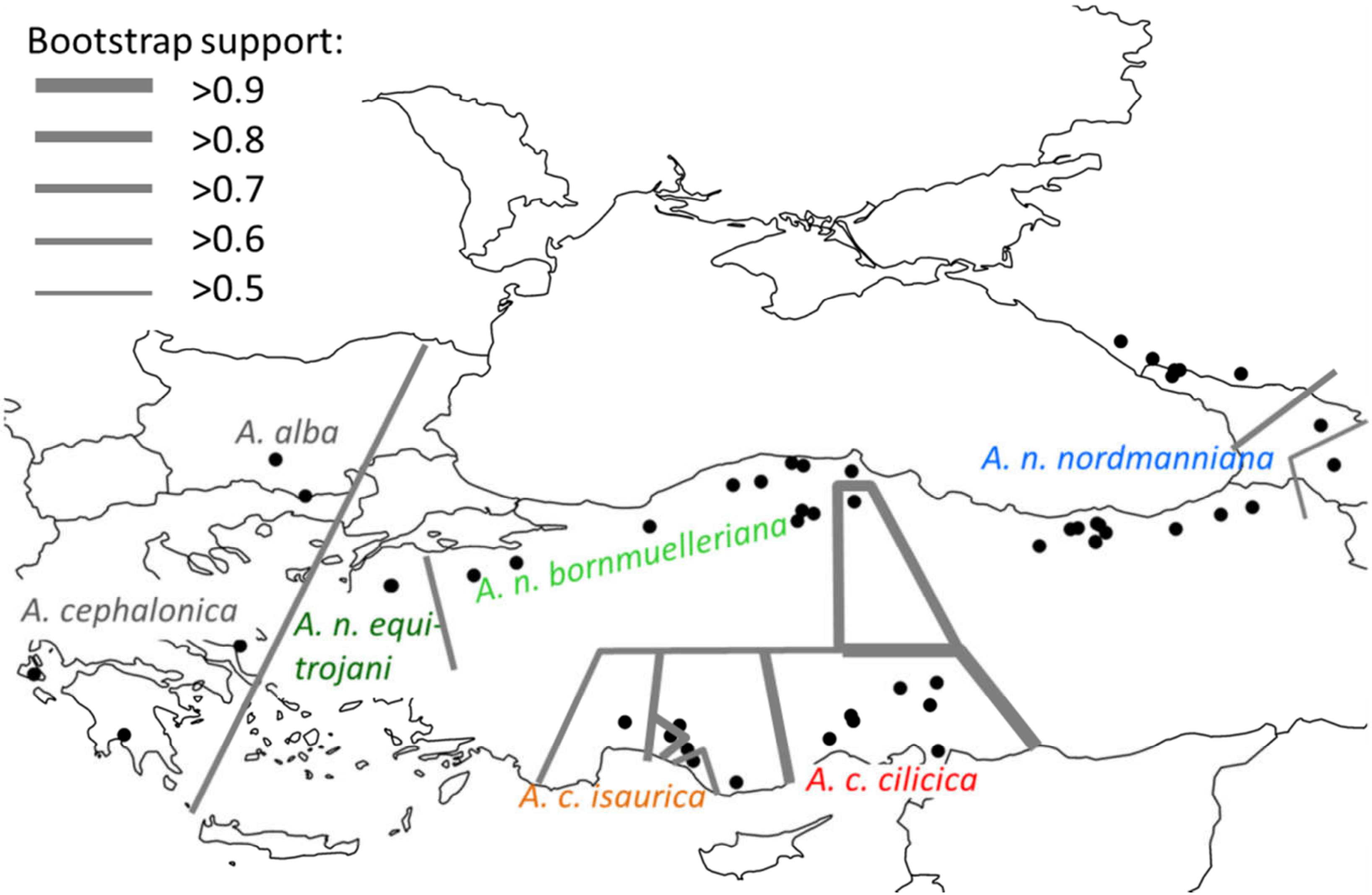

Figure S6 Pre-evaluation of evolutionary scenarios relying on prior distributions of parameters and model checking relying on posterior distributions of parameters for the selected best scenario based on principal component analysis of summary statistics for the tested configurations of the studied Mediterranean fir taxa and the corresponding scenarios

a) *A. bornmuelleriana – A. equi trojani – A. × olcayana*

Prior distribution for the tested scenarios

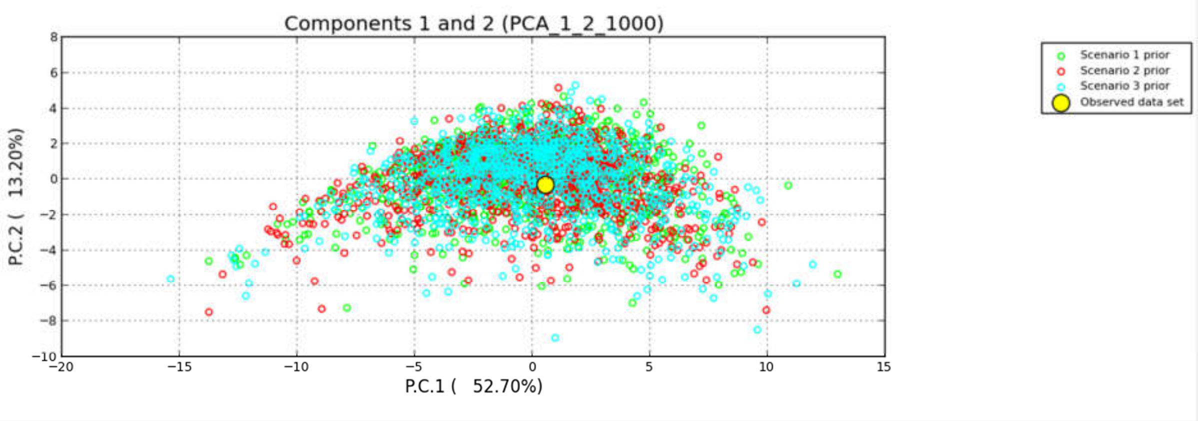

Posterior distribution for the selected best scenario

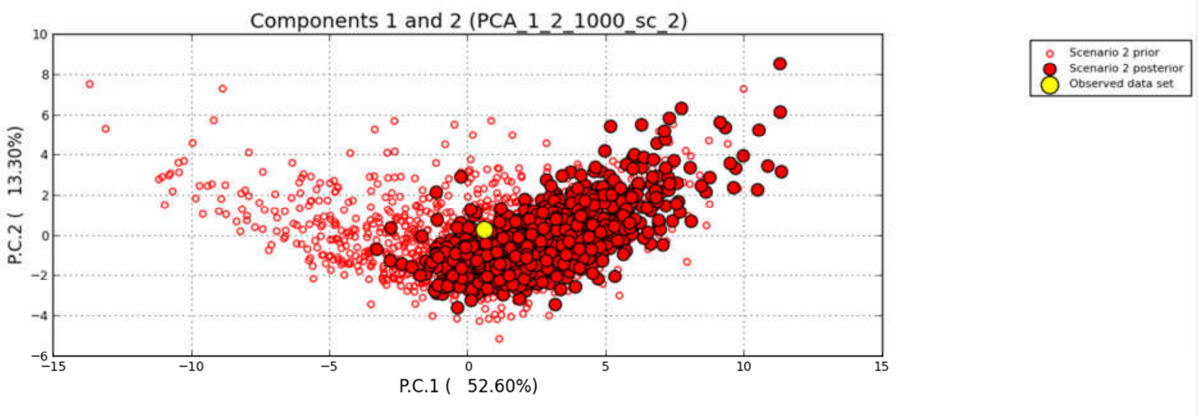

b) *A. bornmuelleriana – A. equi trojani – A. alba*

Prior distribution for the tested scenarios

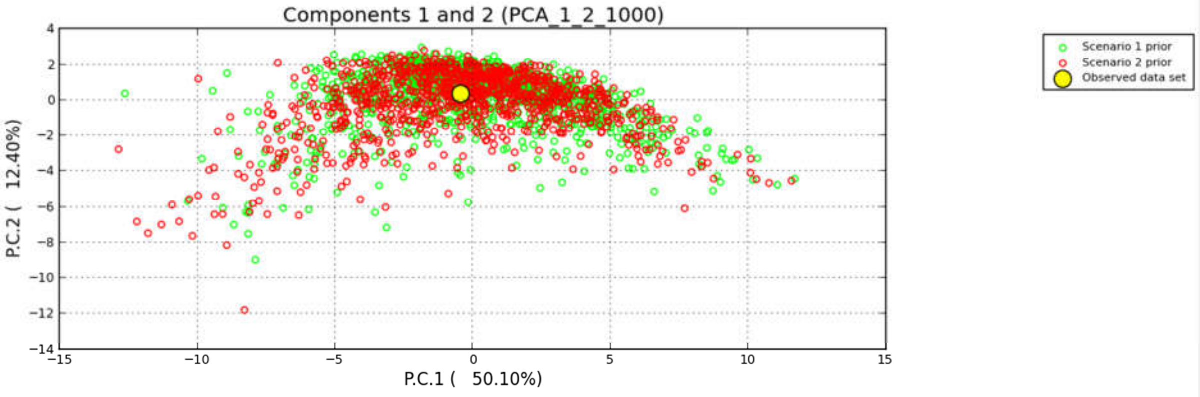

Posterior distribution for the selected best scenario

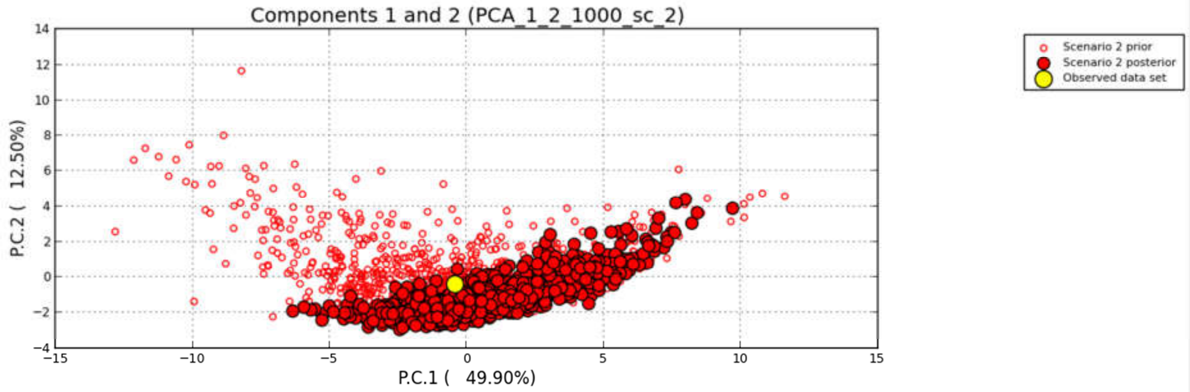

c) *A. nordmanniana* s.l.

Prior distribution for the tested scenarios

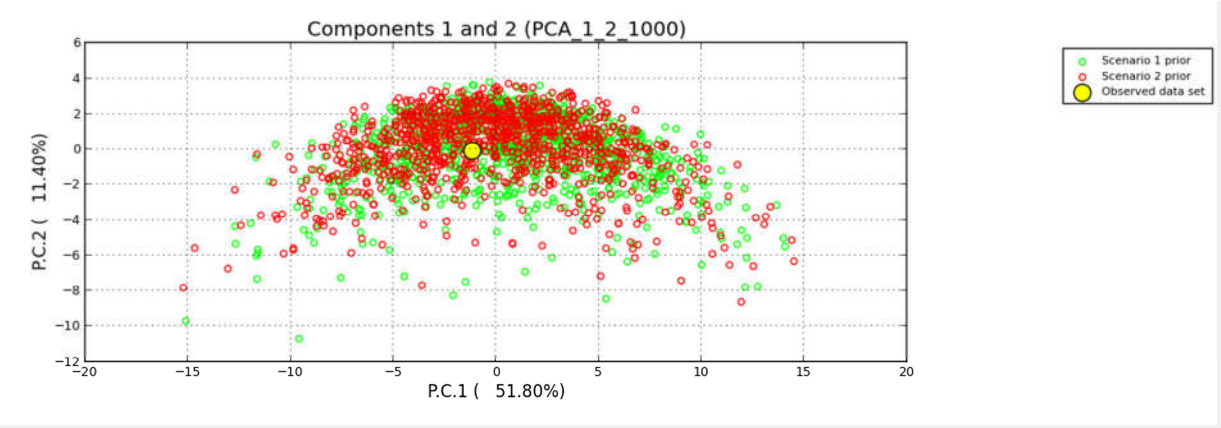

Posterior distribution for the selected best scenario

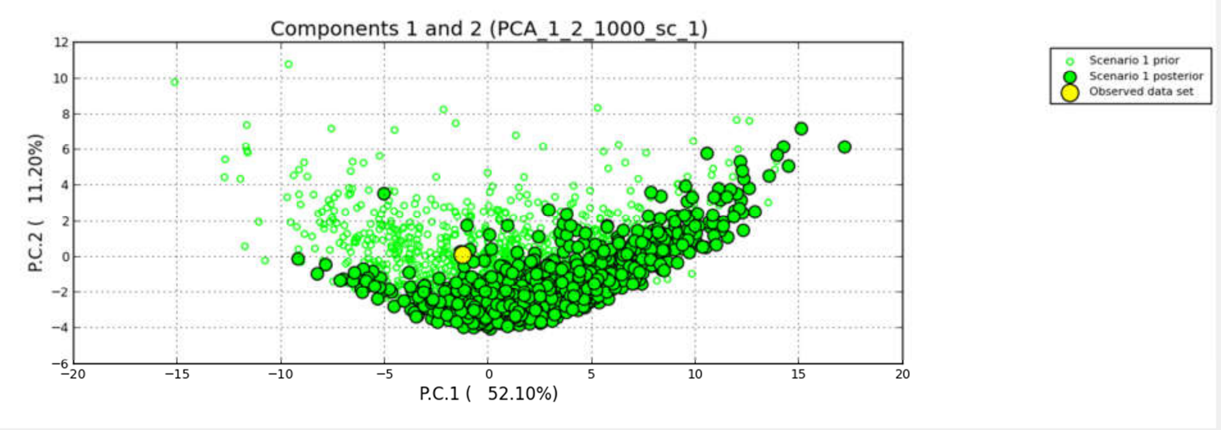

d) *A. cilicica* s.l.

Prior distribution for the tested scenarios

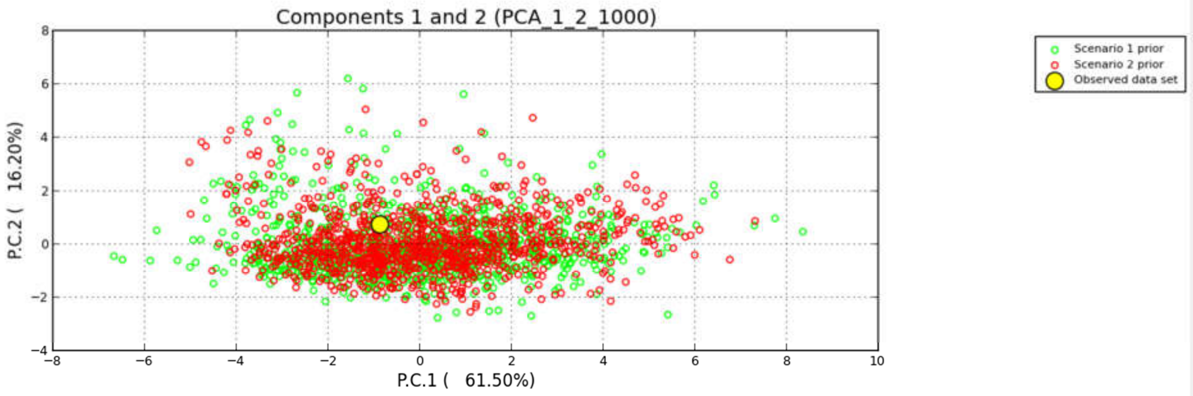

Posterior distribution for the selected best scenario

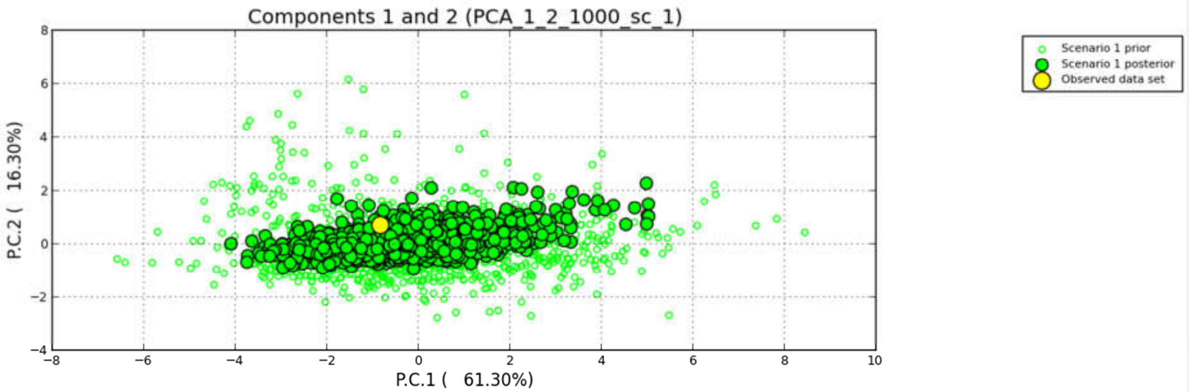

Supplement: Supplementary file 1 [file ECE3-7-10018-s001.pdf]
